# Identification of Survival-Related Genes in Acute Myeloid Leukemia (AML) Based on Cytogenetically Normal AML Samples Using Weighted Gene Coexpression Network Analysis

**DOI:** 10.1155/2022/5423694

**Published:** 2022-09-29

**Authors:** Tingting Chen, Juan Zhang, Yinying Wang, Hebing Zhou

**Affiliations:** Department of Hematology, Beijing Luhe Hospital, Capital Medical University, Beijing, China

## Abstract

The prognosis of acute myeloid leukemia (AML) remains a challenge. In this study, we applied the weighted gene coexpression network analysis (WGCNA) to find survival-specific genes in AML based on 42 adult CN-AML samples from The Cancer Genome Atlas (TCGA) database. Eighteen hub genes (*ABCA*13, *ANXA*3, *ARG*1, *BTNL*8, *C11orf*42, *CEACAM*1, *CEACAM*3, *CHI*3*L*1, *CRISP*2, *CYP*4*F*3, *GPR*84, *HP*, *LTF*, *MMP*8, *OLR*1, *PADI*2, *RGL*4, and *RILPL*1) were found to be related to AML patient survival time. We then compared the hub gene expression levels between AML peripheral blood (PB) samples (*n* = 162) and control healthy whole blood samples (*n* = 337). Seventeen of the hub genes showed lower expression levels in AML PB samples. The gene expression analysis was also done among AML BM (bone marrow) samples of different stages: diagnosis (*n* = 142), posttreatment (*n* = 42), and recurrent (*n* = 12) stages. The results showed a significant increase of *ANXA*3, *CEACM*1, *RGL4*, *RILPL*1, and *HP* in posttreatment samples compared to diagnosis and/or recurrent samples. Transcription factor (TF) prediction of the hub genes suggested *LTF* as the top hit, overlapping 10 hub genes, while *LTF* itself is just one of the hub genes. Also, 3671 correlation links were shown between 128 mRNAs and 209 lncRNAs found in survival time-related modules. Generally, we identified candidate mRNA biomarkers based on CN-AML data which can be extensively used in AML prognosis. In addition, we mapped their potential regulatory mechanisms with correlated lncRNAs, providing new insights into potential targets for therapies in AML.

## 1. Background

The malignant hematologic disease, acute myeloid leukemia (AML), is a heterogeneous clonal disorder of myeloid progenitors that accumulates due to a blockage in their differentiation and infiltration into other organs of the body (mainly the liver and spleen and to a lesser extent the lymph nodes, central nervous system, and testicles), leading to death [1–3]. The pathogenesis of AML is often accompanied by cytogenetic and molecular biological abnormalities. No specific pathogenic factors of AML have been discovered.

Cytogenetically normal acute myeloid leukemia (CN-AML) presents without microscopically detectable chromosomal abnormalities and contributes to approximately 50% of the observed AML cases [4]. Heterogeneity is common within patients with CN-AML. With the advancement of genomics research, molecular genetic analysis has allowed for a more detailed pretreatment assessment of CN-AML prognosis, which can be graded by their molecular genetic characteristics. Many genes are involved in the molecular mechanisms of AML, leading to complexities in AML diagnosis and prognosis. Previous studies identified various DNA and RNA markers as prognostic factors for CN-AML, such as *NPM1* and *CEBPA*, in which mutations have been proposed as good prognostic factors, as well as *PLT3*, *RUNX1*, *ASXL1*, and *TP53*, in which mutations have been considered to be correlated with poor prognosis [4, 5]. Treatment-dependent factors are also important in estimating the prognosis of CN-AML patients. For example, platelet (PLT) counts at diagnosis are proved to be able to predict survival for patients with intermediate-risk AML [6]. Also, in another study, CD45^dim^CD117^+^ phenotypical abnormal cell ratio > 2.055% within 2 weeks after the first complete remission (CR) is considered to be an independent risk factor for recurrence, which also is an adverse factor for relapse-free survival (RFS) and overall survival (OS) in adult AML patients [7]. However, due to the highly variable molecular genetic prognostic yield, prognostic genes of AML require further exploration.

To better understand the complex prognostic gene expression signatures of CN-AML and investigate potential targeted therapies, we performed the weighted gene coexpression network analysis (WGCNA) on the RNA-seq data of adult patients with CN-AML, available from The Cancer Genome Atlas (TCGA). Our study identified survival-specific genes and provided system-level evidence of genetic networks that contribute to the prognosis of adult CN-AML patients. What is more, the survival-specific genes we found based on CN-AML samples also showed prognostic values in AML samples regardless of any clinical characteristics (including age and the existence of chromosomal changes).

## 2. Materials and Methods

### 2.1. Study Design and Data Curation

**Figure 1:**
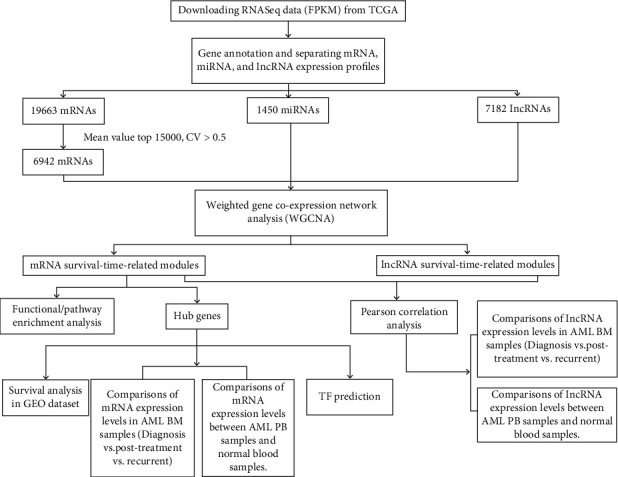
The work flow of this research.

Figure 1 provides a flowchart of the study process. Forty-two adult patients with CN-AML were selected from TCGA database (https://portal.gdc.cancer.gov/) (project TCGA-LAML [8]) for the WGCNA (see the clinical information in Table 1. For more detailed information, please see Table 1S). The sample screening criteria were (a) patients with integral RNA-seq data and clinical trait data, (b) patients who were cytogenetically normal, (c) patients who were deceased and the date of death ≥ 30 days from the date of initial pathologic diagnosis, and (d) the age at diagnosis was ≥18.

To perform the survival analysis among the hub genes obtained after the WGCNA, we chose 148 adult (≥18 years) CN-AML patients with an OS > 30 days from the Gene Expression Omnibus (GEO) database (http://www.ncbi.nlm.nih.gov/geo/; accession number GSE12417 [9], platform GPL96) (for more detailed information, please see Table 2S).

To compare the expression levels of hub genes and correlatedly expressed lncRNAs in AML BM samples of different stages, we chose 196 samples of 163 patients from an independent cohort in the Therapeutically Applicable Research to Generate Effective Treatments (TARGET) database (https://ocg.cancer.gov/programs/target) [10]. The sample screening criteria were primary AML BM samples with RNA expression profiles from diagnosis, posttreatment, or recurrent stages regardless of their clinical characteristics (for more detailed patient information, please see Table 3S).

To compare the expression levels of hub genes and correlatedly expressed lncRNAs between primary AML PB samples at the diagnosis stage and normal whole blood samples, we chose 133 samples from TCGA database (https://portal.gdc.cancer.gov/) and 29 samples from the TARGET (https://ocg.cancer.gov/programs/target) database (samples from TCGA database do not include the 42 CN-AML used for WGCNA). The AML PB sample screening criteria were primary AML PB samples of the diagnosis stage with RNA expression profiles; AML PB samples were selected regardless of their clinical characteristics (see Tables 4S and 5S for the sample details). Also, 337 healthy whole blood samples were selected from the Genotype-Tissue Expression (GTEx) database [11] (https://www.gtexportal.org/home/) to serve as normal controls. The healthy whole blood sample screening criteria were healthy whole blood samples with RNA expression profiles.

### 2.2. Data Preprocessing

We collected the fragments per kilobase of exon model per million (FPKM) mapped reads [12] and standardized the RNA-seq data from the TCGA-LAML project. mRNA, miRNA, and lncRNA expression profiles were separated and annotated according to the GENCODE (v29) database [13]. A total of 19663 mRNA, 1450 miRNA, and 7182 lncRNA expression profiles were obtained. For mRNAs, only the top 15,000 genes (ranked by their mean values) with a coefficient of variation (CV) > 0.5 were selected for subsequent analysis, resulting in 6942 mRNAs. Owing to the constant nature of the updates to TCGA database, we used the survival time of deceased patients, other than OS in the WGCNA to define the survival-related gene modules.

### 2.3. WGCNA

**Figure 2:**
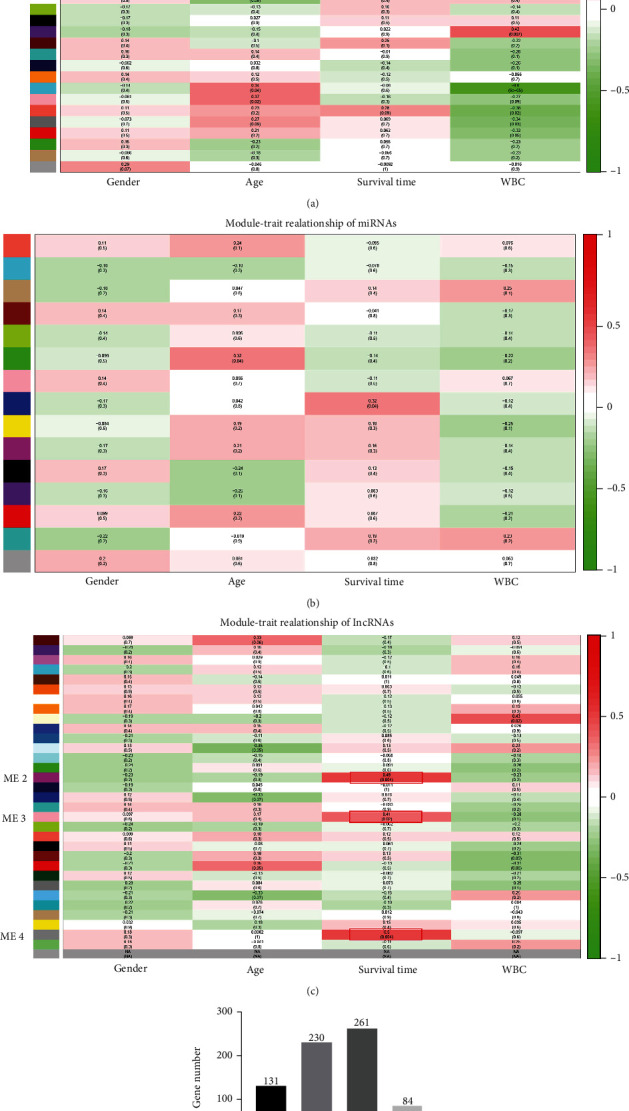
Module-trait associations and gene numbers in the survival time positively related modules. (a–c) The positive and negative correlation coefficients of WGCNA modules and clinical characteristics of mRNAs, miRNAs, and lncRNAs were colored red and green, respectively. Each cell contains the corresponding correlation and *P* value. The more intense red indicates a positive correlation; the more intense green indicates a negative correlation. ME1 module of mRNAs (a), as well as the ME2, ME3, and ME4 modules of lncRNAs (c), showed positive associations with the survival times of the adult CN-AML patients (marked with red frames). (d) Gene numbers in ME1, ME2, ME3, and ME4 modules.

**Figure 3:**
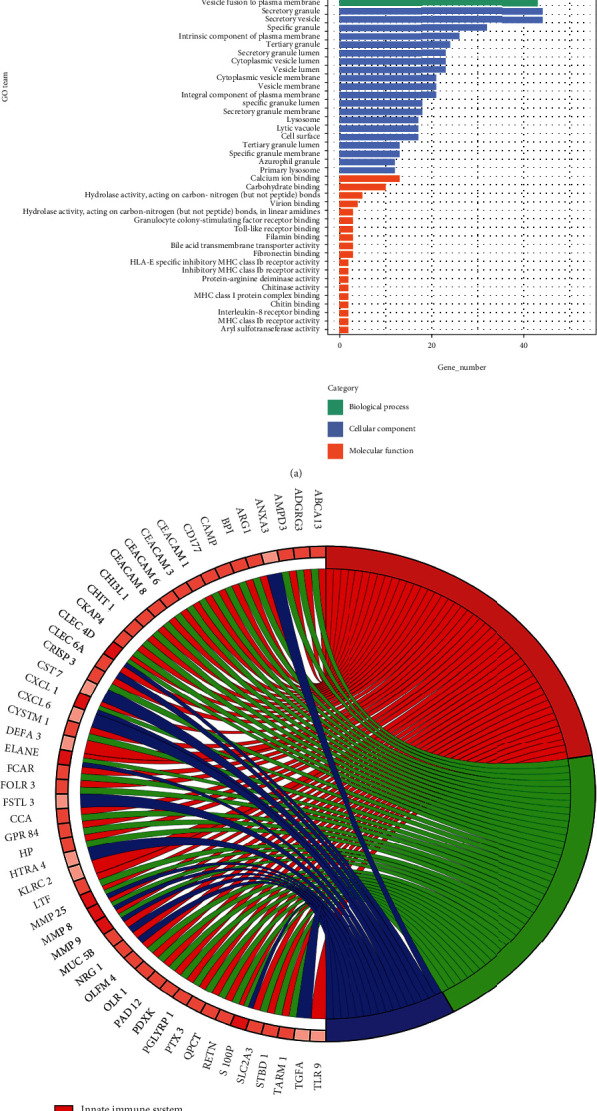
The GO function and pathway enrichment analyses of mRNAs in ME1. (a) Top 20 gene ontology terms with the *Q* value < 0.05 of mRNAs from ME1 module. The *x* axis represents gene number, and the *y* axis represents GO terms. (b) Pathways with the *Q* value < 0.05 and the hit gene number > 10% of the mRNAs from ME1 module. The color shades of the genes represent the numbers of the pathways the genes are enriched in (from 1 to 3 in this figure). The darker the color is, the more pathways the gene is enriched in.

WGCNA was performed on lncRNA, miRNA, and mRNA expression data separately using the R package “WGCNA” [14]. Clinical information of patients including gender, age, white blood cell count (WBC), and survival time was explored to identify the coexpression modules associated with disease progression. First, the expression data were cleaned by removing visible outlier samples (Figure 1S) and genes. Genes of similar expression patterns were divided into modules based on their Euclidean distances (Figures 2S A, 2S C, and 2S E). To construct an unsigned weighted gene network, the proper soft thresholding power beta was chosen, and the coexpression similarity was raised to calculate adjacency. To ensure a scale-free network, the power of the *β* values for mRNAs, miRNAs, and lncRNAs was 5, 4, and 4, respectively (Figure 3S). The adjacency was converted into a topological overlap matrix (TOM), followed by the corresponding dissimilarity calculation. Second, a hierarchical clustering tree of genes, also called a dendrogram, was generated by hierarchical clustering, and the dynamic tree cut was used to identify the coexpression gene modules. Next, the module-trait associations were quantified to identify important modules. The associations of individual genes with the trait of interest were defined by gene significance (GS) as the gene-clinical trait correlation. Also, module membership (MM) was defined to quantify the relevance between module eigengenes and the gene expression profiles. Finally, genes with high GS for interesting traits and high MM in important modules were identified.

### 2.4. Functional and Pathway Enrichment Analysis

The ToppGene database (https://toppgene.cchmc.org/ (accessed on Jul. 30^th^, 2022)) was applied to statistically identify enriched pathways and gene ontologies (GO) [15]. The cut-off value was set to *Q* value < 0.05 [16]. The results were then visualized by using R package “ggplot2” and “GOPlot” [17, 18].

### 2.5. Protein-Protein Interaction (PPI) Network Construction

The online database Search Tool for the Retrieval of Interacting Genes (STRING) (Version 11.0) (https://string-db.org/) was used to construct the PPIs [19], with a combined score > 0.4 as the cut-off criterion. The Cytoscape software (Version 3.7.0) was used for visualization and analysis of the biomolecular interaction networks [20].

### 2.6. Screening of Hub Genes

The cytoHubba plugin of the Cytoscape software was used to identify the hub genes of the interested mRNA modules [21]. Twelve scoring methods were used to screen the hub genes. The methods were Maximum Clique Centrality (MCC), Density of Maximum Neighborhood Component (DMNC), Maximum Neighborhood Component (MNC), Degree, Edge Percolated Component (EPC), BottleNeck, EcCentricity, Closeness, Radiality, Betweenness, Stress, and ClusteringCoefficient. Genes listed in the top 20 ranked nodes by no less than 5 of the scoring methods were identified as the hub genes.

### 2.7. Survival Analysis of Hub Genes in GEO Dataset

The survival analysis based on the hub gene mRNA expression levels and patient OS was analyzed by an online tool, GenomicScape (http://genomicscape.com/) [22]. The probe set with the highest standard deviation (SD) was selected when more than one probe set interrogated the same gene.

### 2.8. Expression Analyses of Hub Genes and lncRNAs among Different Stages of AML BM Samples or between AML PB Samples and Healthy Blood Samples

The expression matrices with RSEM (RNA-Seq by Expectation Maximization) [23] normalized count data of genes in AML BM samples, AML PB samples, and healthy blood samples (the samples were from TCGA, TARGET, and GTEx databases) were obtained from the UCSC XENA database (https://xenabrowser.net/) [24].

### 2.9. Transcription Factor (TF) Prediction for the Hub Genes

TF prediction for the hub genes was done via the ChEA3 (https://amp.pharm.mssm.edu/chea3/) website [25].

### 2.10. Statistical Analysis

The RStudio software (http://www.rstudio.com), Microsoft Excel 2007, the Cytoscape software (Version 3.7.0), and GraphPad Prism 7 were used for all statistical analysis or graphic drawings in this research. *P* values < 0.05 were considered statistically significant [26].

## 3. Results

### 3.1. Key Modules and Survival-Specific Genes Identified by WGCNA

A total of 29, 15, and 33 modules were identified for mRNAs, miRNAs, and lncRNAs, respectively (Figures 2S B, 2S D, and 2S F).

The relationship between each module and the CN-AML clinical information was tested. We found that ME (module eigengene) 1 module of mRNAs, as well as ME2, ME3, and ME4 modules of lncRNAs, showed positive associations with the survival time of adult patients with CN-AML (Figures 2(a)–2(c)), suggesting that ME1, ME2, ME3, and ME4 modules may play a key role in CN-AML patients surviving. The gene numbers in these modules were 131, 230, 261, and 84, respectively (Figure 2(d)).

**Figure 4:**
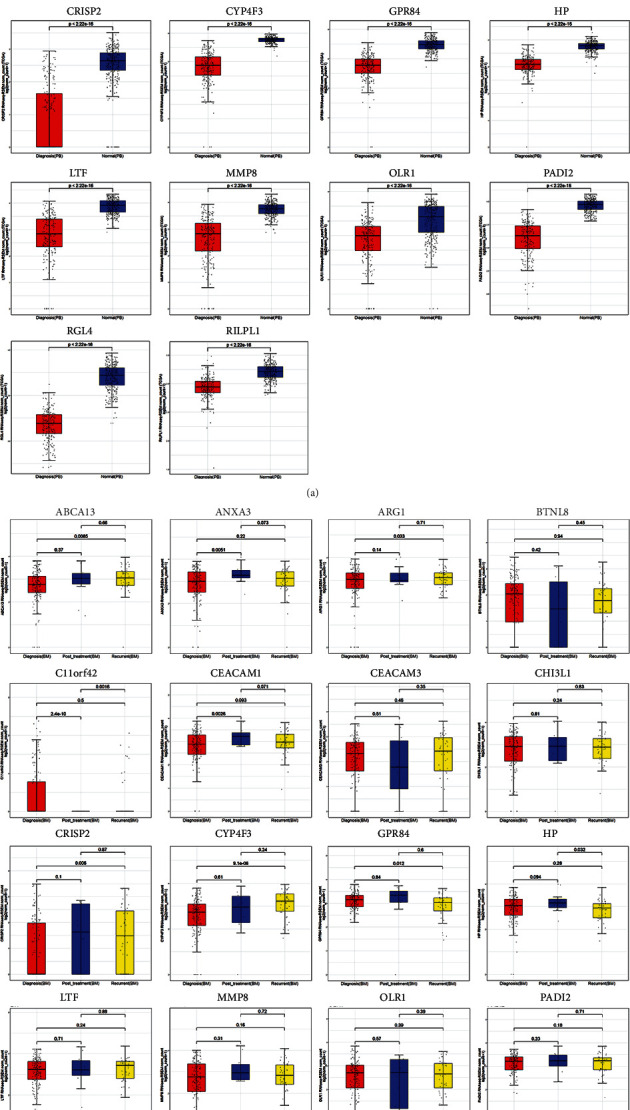
Analyses of hub gene expression levels in AML PB samples, healthy whole blood samples, and AML BM samples of different stages. The lines inside the boxes represent mean values. (a) Comparison of AML PB samples of the diagnosis stage and healthy whole blood samples. (b) Comparison of AML BM samples of diagnosis stage, posttreatment stage, and recurrent stage.

To further explore the association of these four modules with patient survival time, we used GS and MM measures to identify the genes with both high GS for “survival time,” as well as high MM in the selected modules. As shown in Figure 4S, GS and MM were moderately correlated in the ME1 module of mRNAs (cor = 0.57, *P* = 1.2*e* − 12) and the ME3 module of lncRNAs (cor = 0.46, *P* = 4.5*e* − 15) and strongly correlated in ME2 (cor = 0.72, *P* = 4.9*e* − 38) and ME4 (cor = 0.71, *P* = 4*e* − 14) modules of lncRNAs, indicating that genes significantly associated with survival time were also key elements of modules associated with survival time. Thus, we considered genes from the ME1 module of mRNAs, together with those in the ME2, ME3, and ME4 modules of lncRNAs, as survival-specific in adult patients with CN-AML.

### 3.2. Functional/Pathway Enrichment Analysis and PPI Network Establishment

To explore the survival-specific protein-coding genes, the GO analysis of BP, MF, and CC, as well as pathway analyses, was performed on the 131 mRNAs of the ME1 module. The top 20 GO terms of each category are shown in Figure 3(a) and listed in Table 6S. The biological progress (BP) analysis revealed that the survival-specific protein-coding genes were notably enriched in cell activation, leukocyte activation, immune effector process, secretion, myeloid leukocyte activation, and like. The cell component (CC) analysis showed that the ME1 genes were highly concentrated in the compositions of secretory granule, secretory vesicle, specific granule, etc. The molecular function (MF) showed that the ME1 genes were mainly related to calcium ion binding, carbohydrate binding, and so on. The innate immune system, neutrophil degranulation, and ensemble of genes encoding ECM- (extracellular matrix-) associated proteins (including ECM-affiliated proteins, ECM regulators, and secreted factors) are the top three hits in the pathway analysis with the hit gene number > 10% of the ME1 mRNAs (Figure 3(b) and Table 6S).

**Figure 5:**
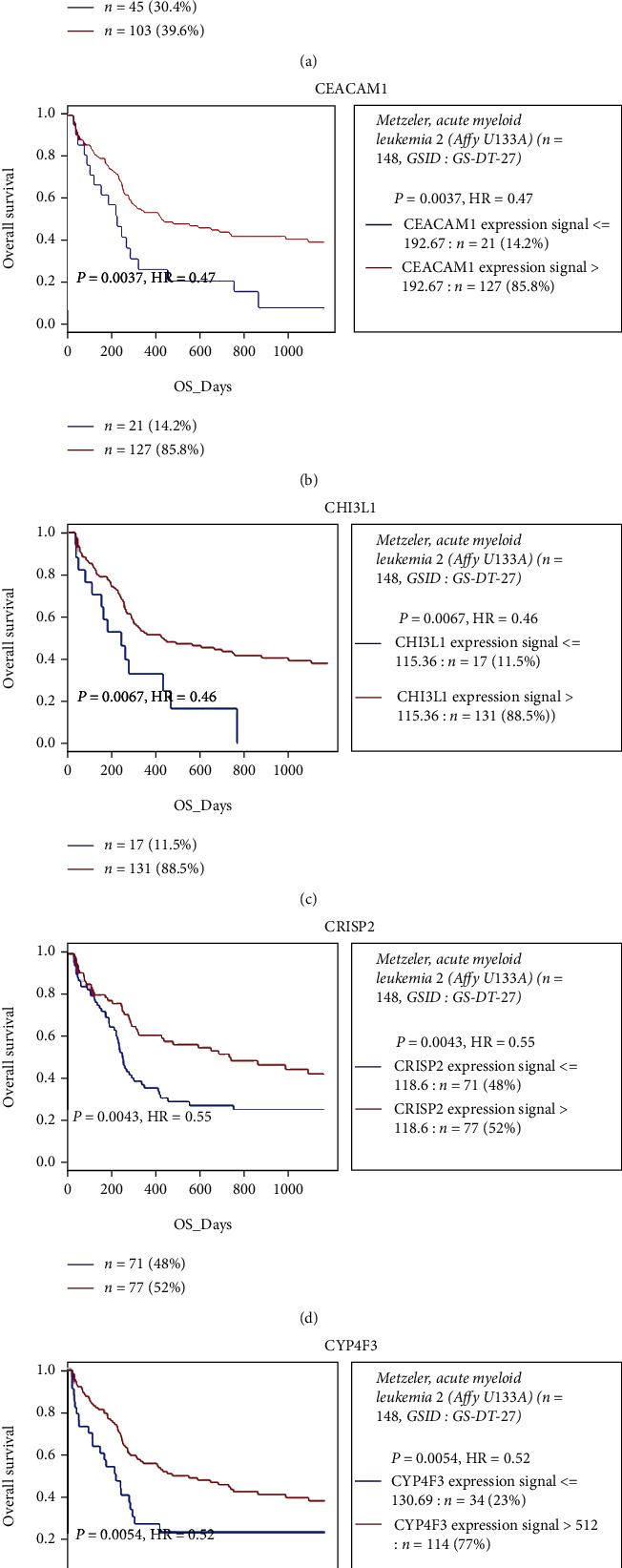
Prognostic values of the mRNA expression of *ARG*1 (a), *CEACAM*1 (b), *CHI3L*1 (c), *CRISP*2 (d), and *CYP*4*F*3 (e) in 148 adult CN-AML patients of the GSE12417 dataset from the GEO database.

Next, we established a PPI network of the ME1 mRNAs recognized in STRING, as shown in Figure 5S.

### 3.3. Hub Gene Identification and Validation

We obtained 18 hub genes from 131 mRNAs of the ME1 module by the method we described above, using the cytoHubba plugin of the Cytoscape software. These were *ABCA13*, *ANXA3*, *ARG1*, *BTNL8*, *C11orf42*, *CEACAM1*, *CEACAM3*, *CHI3L1*, *CRISP2*, *CYP4F*3, *GPR84*, *HP*, *LTF*, *MMP8*, *OLR1*, *PADI2*, *RGL4*, and *RILPL1*.

**Figure 6:**
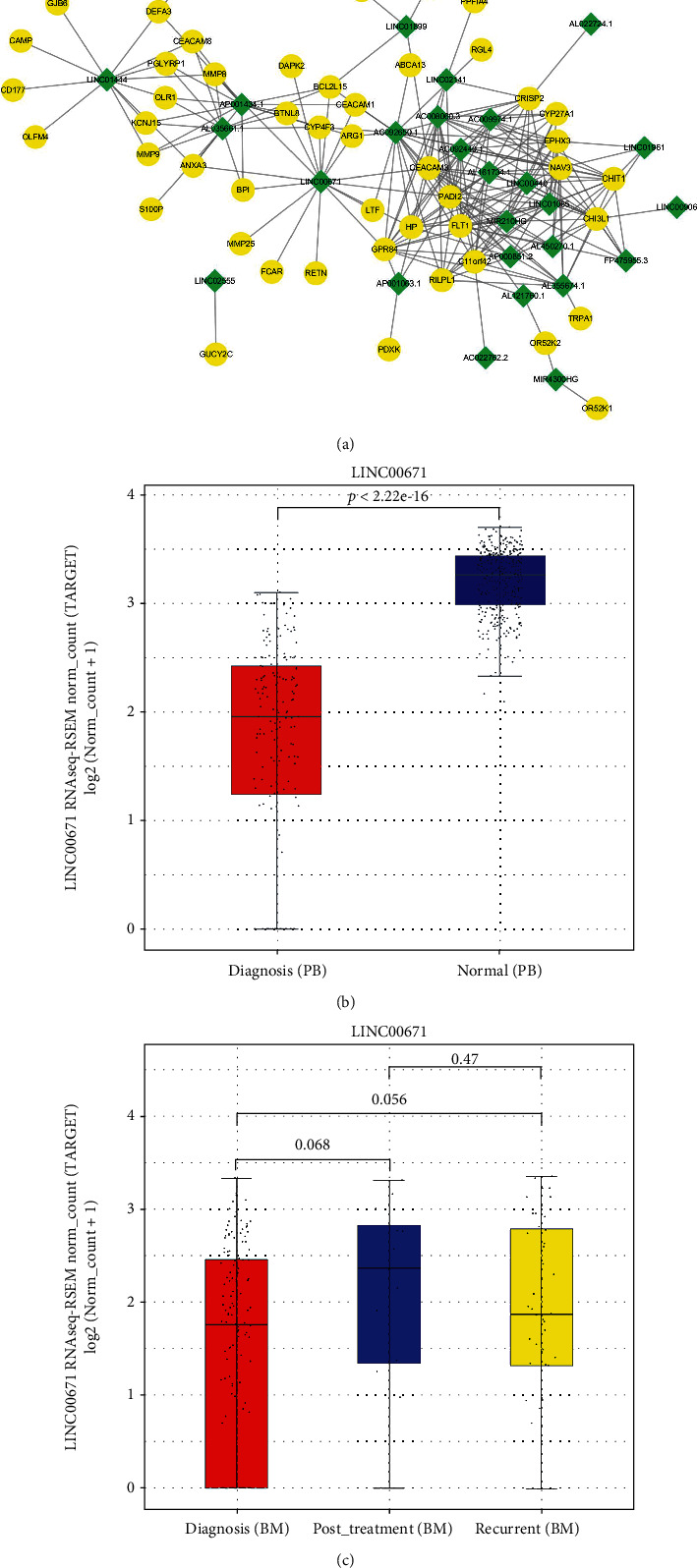
Pearson's correlation analysis between mRNAs and lncRNAs. (a) Coexpression network of 127 mRNAs and 28 lncRNAs with a |*R*| > 0.8 (*P* < 0.05) based on Pearson's correlation analysis. Yellow round nodes indicate mRNAs, and green diamond nodes indicate lncRNAs. (b) The expression analysis of *LINC*00671 between AML PB samples and healthy whole blood samples. The lines inside the boxes represent mean values. (c) The expression analysis of *LINC*00671 among AML BM samples from diagnosis stage, posttreatment stage, and recurrent stage. The lines inside the boxes represent mean values.

The expression levels of the hub genes in PB samples of primary AML patients from the diagnosis stage and healthy whole blood samples were analyzed. We separately compared TCGA AML PB samples vs. GTEx healthy samples (Figure 6S A) and TARGET PB samples vs. GTEx healthy samples (Figure 6S B). Also, we integrated AML PB samples from TCGA and TARGET databases and compared them with GTEx healthy samples (Figure 4(a)). In whichever analyzing way, we observed that 17 of the 18 hub genes (except *C11orf42*) had a decreased expression level in AML PB samples compared to healthy samples (*P* < 0.05).

Next, we compared the hub gene expression levels in primary AML BM samples of three different stages: diagnosis stage, posttreatment stage, and recurrent stage (all samples here are from the TARGET database). From Figure 4(b), we observed some interesting changes in hub gene expression among samples of different stages. *ANXA3* and *CEACM1* are significantly upregulated in posttreatment samples compared to diagnosis samples (*P* < 0.05). *RGL4* and *RILPL1* are significantly upregulated in posttreatment samples compared to both diagnosis and recurrent samples (*P* < 0.05). *ABCA13*, *ARG1*, *CRISP2*, and *CYP4F3* showed higher expression levels in recurrent samples than in diagnosis samples (*P* < 0.05). Also, the expression level of *HP* in posttreatment samples is significantly higher than that in recurrent samples (*P* < 0.05).

Survival analysis of the 18 hug genes was then performed in an independent cohort of 148 patients with CN-AML from the GEO database, using GenomicScape. We found that higher expression levels of 5 genes, *ARG1*, *CEACAM1*, *CHI3L1*, *CRISP2*, and *CYP4F3*, were significantly correlated with a longer OS (*P* < 0.05) (Figure 5).

We then predicted TFs for the 18 hub genes by ChEA3 website. The top 10 TFs were listed in Table 2. From the results, we noticed that *Lactotransferrin* (*LTF*) ranked the first place with the lowest mean rank [25] and the most overlapping genes (*CEACAM3*, *CEACAM1*, *ANXA3*, *ARG1*, *CYP4F3*, *CHI3L1*, *PADI2*, *RGL4*, *MMP8*, and *ABCA13*). Also, *LTF* is just one of our 18 hub genes.

### 3.4. Pearson's Correlation Analysis between mRNAs and lncRNAs

To explore the potential regulatory mechanisms linking the lncRNAs of modules ME2, ME3, and ME4 with the mRNAs of module ME1, we performed Pearson's correlation analysis based on their expression data from 42 TCGA samples. The 128 mRNAs and 209 lncRNAs formed 3671 correlation links (|*R*| > 0.5, *P* < 0.05). In particular, 127 mRNAs and 28 lncRNAs formed 224 very strong [27] correlation links with an |*R*| > 0.8 (*P* < 0.05) (Figure 6(a), Table 7S). The top 2 lncRNAs having the most linked mRNAs are *AC*092650.1 and *LINC*00671, linked to 19 and 17 mRNAs, respectively. However, there are no studies about *AC*092650.1 yet. But *LINC*00671 has been reported serving as an anticarcinogenic role in various kinds of cancers [28–31]. We analyzed the expression level of *LINC*00671 in AML PB samples and normal peripheral blood samples. Notably, we found that LINC00671 showed decreased expression in AML PB samples compared to healthy blood samples (Figure 6(b)). No significant expression differences were shown among the samples of diagnosis, posttreatment, and recurrent stages, but we can see a trend of increased expression in the posttreatment group compared to the other 2 groups (Figure 6(c)). Since we only have 12 posttreatment samples here, maybe there will be statistical significance when more samples are available.

## 4. Discussion

Patients with AML without chromosomal changes are diagnosed as CN-AML [32]. Having no microscopically detectable chromosomal abnormalities in leukemic blasts makes CN-AML cytogenetically uniform and provides a perfect platform for AML biomarker recognition. Here, we used the WGCNA methodology to identify the prognosis-related biomarkers of AML on the basis of RNA-seq and clinical trait data of CN-AML samples.

WGCNA, an algorithm for a scale-free network introduced in 2005, has been used to propose candidate therapeutic targets or predict diagnosis, classification, progression, or prognosis in various types of cancers [33–37]. As an effective bioinformatics tool for outlining gene correlation patterns, WGCNA not only identifies but also weights gene connections by the association between sample expression profiles and clinical features, for the construction of more accurate and complete gene networks [14]. lncRNAs play multifaceted roles in both health and disease, including cancer [38]. One assumption of the lncRNA functional mechanism is the competitive endogenous RNA (ceRNA) hypothesis. This suggests that lncRNAs may nullify miRNA, subsequently upregulate the expression of downstream miRNA target genes [39]. This hypothesis has been experimentally substantiated in various types of cancers, including hematological malignancies [40–44]. Nowadays, there are more and more studies involving applying WGCNA in AML-related analysis published in journals of different levels [45–50], which proves the recognition of this algorithm to some extent. However, there is no study aimed at finding AML survival-specific biomarkers using the WGCNA methodology based on adult CN-AML data by far. Moreover, our study not only is limited to the expression of the mRNA level but also includes miRNA, and lncRNA gene expression data (though no AML characteristic-related miRNA modules were found in our study, which probably means that the miRNA expression profile alone is not capable enough to connect with AML clinical characteristics independently). A total of 19663 mRNAs, 1450 miRNAs, and 7182 lncRNAs were included in our analysis. Based on clinical features (gender, age, survival time, and white blood cell count (WBC)), we identified 1 prognosis-related mRNA module (ME1 module of 131 mRNAs) and 3 lncRNA modules (ME2, ME3, and ME4 modules of 230, 261, and 84 lncRNAs, respectively) from the RNA-seq data and clinical trait data of 42 adult patients with CN-AML that matched our screening criteria.

After constructing a PPI network of 131 mRNAs and mRNA-lncRNA network carried out by Pearson's correlation analysis, we used the cytoHubba plugin of Cytoscape software to find hub genes. CytoHubba provides 12 topological analysis methods, which are MCC, DMNC, MNC, Degree, EPC, BottleNeck, EcCentricity, Closeness, Radiality, Betweenness, Stress, and ClusteringCoefficient, to rank nodes in a network by the network features [51]. These nodes screened for 18 hub genes in our study.

In expression analyses of the hub genes in different cohorts of AML samples and healthy whole blood samples, 17 of the 18 hub genes showed higher expression levels in AML PB samples than in healthy whole blood samples. Nine genes showed higher expression levels in AML BM samples in the posttreatment stage, compared to the diagnosis and recurrent stages. These results were consistent with our expectation of prognostic values of these genes. And it also proved that these potential biomarkers extracted based on CN-AML sample data may be extensively applicable to all kinds of AML samples, regardless of clinical traits. Also, survival analysis of the 18 hub genes in 148 GEO CN-AML patients showed the correlation of higher expression levels of *ARG1*, *CEACAM1*, *CHI3L1*, *CRISP2*, and *CYP4F3* with a longer OS.

In the 18 hub genes, *CEACAM1*, *CRISP2*, and *CYP4F3* showed their strong competitiveness in both expression analyses (AML PB samples vs. healthy blood samples and AML BM samples posttreatment stage vs. diagnosis/recurrent stages) and the survival analysis. They can be key study genes in our further research. Their relationship with tumor progression has been reported in previous studies. *Carcinoembryonic antigen-related cell adhesion molecule 1* (*CEACAM1*) mediates the direct interaction between tumor and immune cells as a cell-cell communication molecule [52]. It has been proved to be a tumor suppressor or biomarker in cancers of different primary sites, including the liver, lung, breast, prostate, stomach, and ovary [53–57], while its role in AML remains to be investigated. *Cysteine-rich secretory protein-2* (*CRISP2*) has been reported to be less expressed in high-grade squamous intraepithelial lesions than in other histological grades, making it a novel biomarker for the detection of cervical cancer [58]. *Cytochrome P450 family 4 subfamily F member 3* (*CYP4F3*) has been reported to have good diagnostic values for osteosarcoma [59], and a potentially functional SNP in *CYP4F3* (rs4646904) may contribute to the etiology of lung cancer [60]. Mizukami et al. proved that *CYP4F3A* was upregulated in all-trans-retinoic acid- (ATRA-) treated AML cell line HL-60 [61].

*LTF* (also known as *LF*) was predicted to be a transcription factor to 10 of the 18 hub genes. It is a member of the transferrin family of genes, and its protein product is found in the secondary granules of neutrophils. Its relationship with various types of tumors including AML has been widely reported. Back in 1988, Davey et al. reported a quantitative decrease in *LTF* staining in AML and myelodysplasia, which supports the concept that abnormal neutrophils and bands are derived from a malignant clone of myeloid precursor cells [62] and also is consistent with our expectations for *LTF* to be a candidate biomarker for AML prognosis.

The pathway enrichment analysis suggested innate immune system, neutrophil degranulation, and ensemble of genes encoding ECM-associated proteins (including ECM-affiliated proteins, ECM regulators, and secreted factors) as the top three hits with the hit gene number > 10% of the ME1 mRNAs (35.11%, 30.53%, and 12.21%, respectively). The innate immune system has been widely reported to be closely related to various kinds of cancers including AML [63, 64]. Neutrophil degranulation has been reported to be enriched with differentially expressed genes between *DNA methyltransferase 3 alpha* (*DNMT3A*) mutation positive and negative AML samples (*DNMT3A* is associated with poor prognosis and appeared to be a potential biomarker) [65]. ECM-associated proteins have been proven to play a functional role in the progression and metastasis of many kinds of cancers, including breast cancer, prostate cancer, and neurofibroma [66–68]. EMC-associated proteins have also been reported to be related to disease development and therapy in AML. Wang et al. claim that the ECM-receptor interaction is an important PD-L1 downstream pathway, which regulates cell proliferation and apoptosis in AML [69]. Berdel et al. suggest that ECM-targeted IL-2 combined with anti-CD33 immunotherapy can be used in posttransplant AML relapse [70].

*LINC00671* is one of the lncRNAs revealing a high expression correlation with mRNAs in our PPI network. It was previously found to be a tumor suppressor in multiple cancers including renal cell cancer, pancreatic cancer, and papillary thyroid tumor by inhibiting the growth and metastasis of cancer cells [26–29]. Although there are no studies about it in hematological malignancies yet, we found its significantly higher expression level in AML PB samples compared to healthy blood samples. Also, a trend can be observed that it could be upregulated in posttreatment AML BM samples than diagnosis or recurrent ones. Further lab experiments are needed to prove its cancer suppressor effect or potential biomarker role in AML.

There are previous studies investigating AML based on the WGCNA method. Wiggers et al. [71] identified clusters of genes selectively correlated to relapse risk in patients of distinct AML subtypes by applying WGCNA on mRNAs in 36 AML samples. Also, Ye et al. analyzed the differentially expressed genes between primary AML samples and relapsed samples applying the WGCNA method and identified genes associated with both relapse and overall survival. These studies show the usefulness of the WGCNA method in finding the relationship between gene expression profile and AML prognosis. Also, one study previously analyzed the survival-specific lncRNAs in 27 underage patients with CN-AML [72]. However, none of the previous studies performed a complete WGCNA on the mRNA, miRNA, and lncRNA expression data, and neither of them suggested the possibility that biomarkers found based on CN-AML data may be applicable to all AML samples.

Admittedly, this work was limited by the sample size and statuses of our WGCNA—42 samples (deceased patients only) were included. More comprehensive studies of larger sample sizes should be performed in the future. Additionally, our study was a bioinformatics analysis. The mRNAs and their potential regulatory lncRNAs identified in this study for their prognostic values should be further investigated by in-depth mechanical approaches such as RT-PCR validation and gene function experiments. To use these results in clinical prognosis prediction, prediction models would be constructed, and PCR-based quantifications might be used in risk grading of adult AML patients.

## 5. Conclusions

In this study, we identified AML survival-specific mRNAs and lncRNAs using the WGCNA methodology based on CN-AML data. Eighteen mRNAs were screened as hub genes of the survival-specific mRNAs. Expression analyses in different cohorts of AML samples revealed 17 of the hub genes (*ABCA13*, *ANXA3*, *ARG1*, *BTNL8*, *C11orf42*, *CEACAM1*, *CEACAM3*, *CHI3L1*, *CRISP2*, *CYP4F*3, *GPR84*, *HP*, *LTF*, *MMP8*, *OLR1*, *PADI2*, *RGL4*, and *RILPL1)* were downregulated in AML PB samples compared to healthy whole blood samples; *ANXA3*, *CEACM1*, *RGL4*, *RILPL1*, and *HP* showed increased expression levels in AML BM samples of the posttreatment stage compared to the diagnosis and/or recurrent stage. Also, the expression levels of *ARG1*, *CEACAM1*, *CHI3L1*, *CRISP2*, and *CYP4F3* were demonstrated to be positively correlated with OS in an independent cohort. One of the hub genes, *LTF*, appeared on top of the TF prediction list, overlapping 10 hub genes. lncRNA-mRNA networks were constructed to exhibit the possible genetic regulatory mechanisms of adult CN-AML. *LINC00671*, which was linked to 17 mRNAs, has been widely reported as a tumor suppressor in various solid tumors. Clearly, this study identified the prognosis-specific biomarkers and the potential lncRNA-related regulatory mechanisms in AML. Our findings suggest CN-AML samples as good sources to investigate the relationship of RNA profiles and AML prognosis, and also provide a necessary groundwork for further exploration of the function and potential applications of these biomarkers as therapeutic targets for AML.

## Figures and Tables

**Table 1:** Clinical information of 42 adult CN-AML patients selected from TCGA database for WGCNA analysis.

TCGA Datasets	
Variables	Case number (*N* = 42)
Age (21-88 years)	
<60	21
>=60	21
Gender	
Female	22
Male	20
FAB	
M0	3
M1	10
M2	11
M3	0
M4	13
M5	4
M6	0
M7	1
WBC/×10^9^/L, median (range)	
32.5(1-203)	
BM blast/%, median (range)	
71(0-98)	
Survival time/days, median (range)	
320(30-1706)	

WBC, white blood cell count; BM, bone marrow; FAB, French–American–British classification systems.

**Table 2:** Top 10 predicted transcription factors (TFs) for the hub genes.

Rank	TF	Score	Library	Overlapping_genes
1	LTF	1	ARCHS4 coexpression, 1; GTEx coexpression, 1	CEACAM3, CEACAM1, ANXA3, ARG1, CYP4F3, CHI3L1, PADI2, RGL4, MMP8, ABCA13
2	CREB5	34.67	ARCHS4 coexpression, 30; Enrichr queries, 46; GTEx coexpression, 28	CEACAM3, ANXA3, CYP4F3, RGL4
3	CREB3L3	39.33	ARCHS4 coexpression, 5; Enrichr queries, 106; GTEx coexpression, 7	BTNL8, CEACAM1, ARG1, CYP4F3, HP
4	NFE4	51	GTEx coexpression, 51	CEACAM3, RGL4
5	NR1H4	53.33	ARCHS4 coexpression, 40; Enrichr queries, 80; GTEx coexpression, 40	CEACAM1, ARG1, CYP4F3, HP
6	ATF5	54.67	ARCHS4 coexpression, 50; Enrichr queries, 108; GTEx coexpression, 6	ARG1, ANXA3, CYP4F3, HP
7	ZNF438	66.33	ARCHS4 coexpression, 64; Enrichr queries, 104; GTEx coexpression, 31	CEACAM3, ANXA3, GPR84, RGL4
8	TBX10	68.67	ARCHS4 coexpression, 35; Enrichr queries, 66; GTEx coexpression, 105	BTNL8, CEACAM1, PADI2
9	HNF4A	73	Literature ChIP-seq, 67; ARCHS4 coexpression, 7; ENCODE ChIP-seq, 18; Enrichr queries, 90; ReMap ChIP-seq, 104; GTEx coexpression, 152	BTNL8, CEACAM1, ARG1, CYP4F3, HP
10	NR1I2	81.75	Literature ChIP-seq, 13; ARCHS4 coexpression, 2; Enrichr queries, 144; GTEx coexpression, 168	BTNL8, CEACAM1, ARG1, CYP4F3, HP

## Data Availability

The datasets generated during and/or analyzed during the current study are available in the online database TCGA (https://portal.gdc.cancer.gov/), TARGET (https://ocg.cancer.gov/programs/target), GTEx (https://www.gtexportal.org/home/), and GEO (http://www.ncbi.nlm.nih.gov/geo/).

## Abbreviations

AML: Acute myeloid leukemia
BM: Bone marrow
BP: Biological process
CC: Cellular component
CEACAM1: Carcinoembryonic antigen-related cell adhesion molecule 1
CN-AML: Cytogenetically normal acute myeloid leukemia
CR: Complete remission
CRISP2: Cysteine-rich secretory protein 2
CV: Coefficient of variation
CYP4F3: Cytochrome P450 family 4 subfamily F member 3
DNMT3A: DNA methyltransferase 3 alpha
ECM: Extracellular matrix
FPKM: Fragments per kilobase of exon model per million
GEO: Gene Expression Omnibus
GO: Gene ontology
GS: Gene significance
GTEx: Genotype-Tissue Expression
LTF: Lactotransferrin
ME: Module eigengene
MF: Molecular function
MM: Module membership
NCCN: National Comprehensive Cancer Network
OS: Overall survival
PB: Peripheral blood
PPI: Protein-protein interaction
RSEM: RNA-Seq by Expectation Maximization
SD: Standard deviation
STRING: Search Tool for the Retrieval of Interacting Genes
TARGET: Therapeutically Applicable Research to Generate Effective Treatments
TCGA: The Cancer Genome Atlas
TF: Transcription factor
TOM: Topological overlap matrix
WBC: White blood cell count
WGCNA: Weighted gene coexpression network analysis.

## Supplementary Materials

Supplementary 1 Figure 1S: outlier sample removal for mRNAs (A), miRNAs (B), and lncRNAs (C) WGCNA.

Supplementary 2 Figure 2S: relationship between clinical traits and sample dendrogram, based on the expression data of mRNAs (A), miRNAs (C), and lncRNAs (E). Clustering dendrograms of mRNAs (B), miRNAs (D), and lncRNAs (F).

Supplementary 3 Figure 3S: analysis of network topology for various soft-thresholding powers in mRNAs (A), miRNAs (B), and lncRNAs (C).

Supplementary 4 Figure 4S: scatterplots of GS for survival time *vs*. MM in selected survival-specific module ME1 (A), ME2 (B), ME3 (C), and ME4 (D).

Supplementary 5 Figure 5S: the protein-protein interaction (PPI) network of mRNAs in ME1.

Supplementary 6 Figure 6S: analyses of hub gene expression levels in primary AML PB samples from diagnosis stage and healthy whole blood samples.

Supplementary 7 Table 1S: detailed clinical information for the 42 CN-AML samples from TCGA database.

Supplementary 8 Table 2S: detailed clinical information for the 148 CN-AML samples from GEO dataset (GSE12417, GPL96).

Supplementary 9 Table 3S: detailed clinical information for the 163 AML patients from TARGET database.

Supplementary 10 Table 4S: detailed clinical information for the 133 AML PB samples from TCGA database.

Supplementary 11 Table 5S: detailed clinical information for the 29 AML PB samples from TARGET database.

Supplementary 12 Table 6S: top 20 GO terms and enriched pathways of the 131 mRNAs in ME1 module.

Supplementary 13 Table 7S: 127 mRNAs of ME1 and 28 lncRNAs of ME2, ME3, and ME4 formed 224 correlation links with an |*R*| > 0.8 (*P* < 0.05).
