# Supplementary figures and images for: Identification of Survival-Related Genes in Acute Myeloid Leukemia (AML) Based on Cytogenetically Normal AML Samples Using Weighted Gene Coexpression Network Analysis
Source: Dis Markers. 2022 Sep 29;2022:5423694. doi: 10.1155/2022/5423694 (PMC9537620; doi:10.1155/2022/5423694)

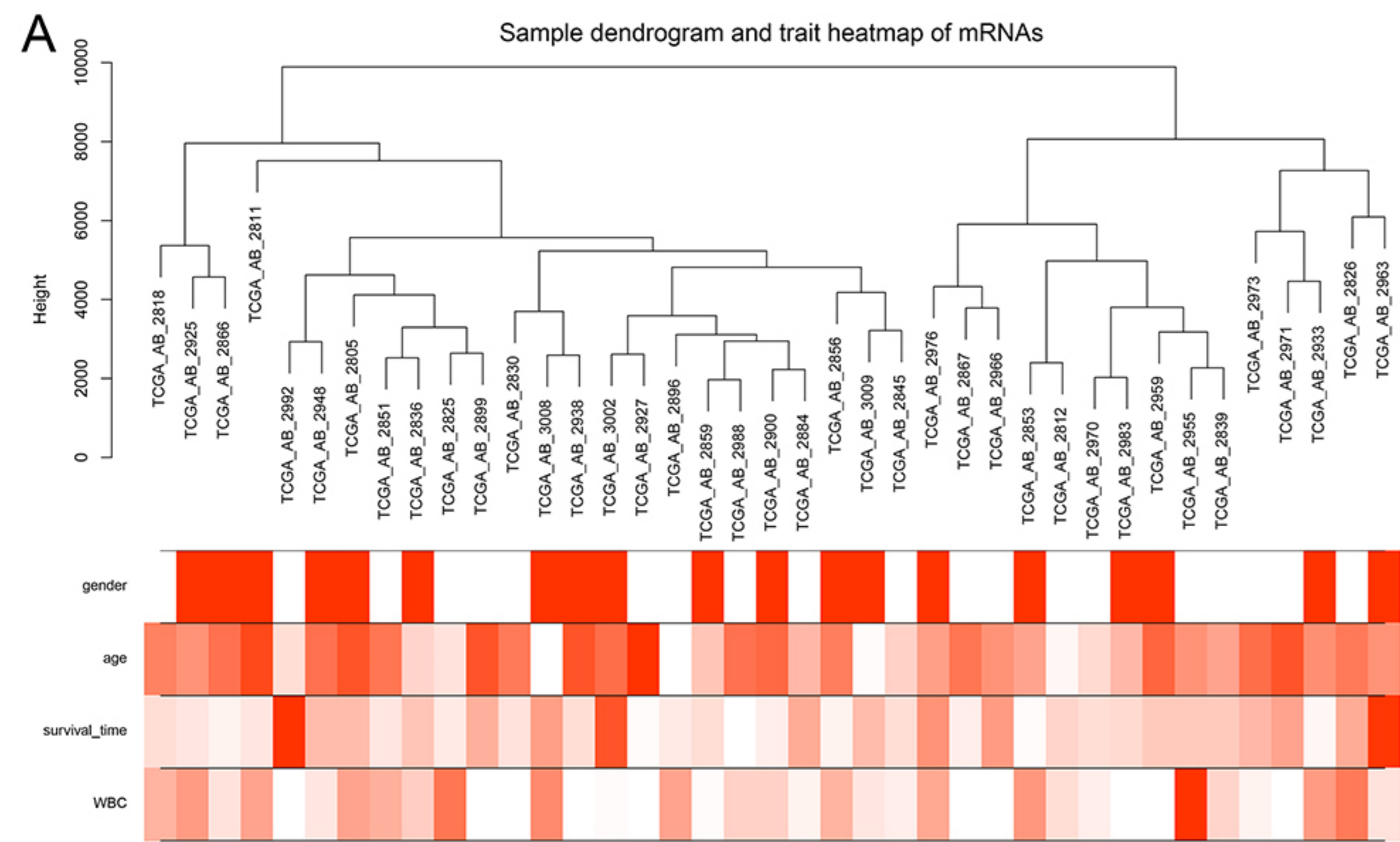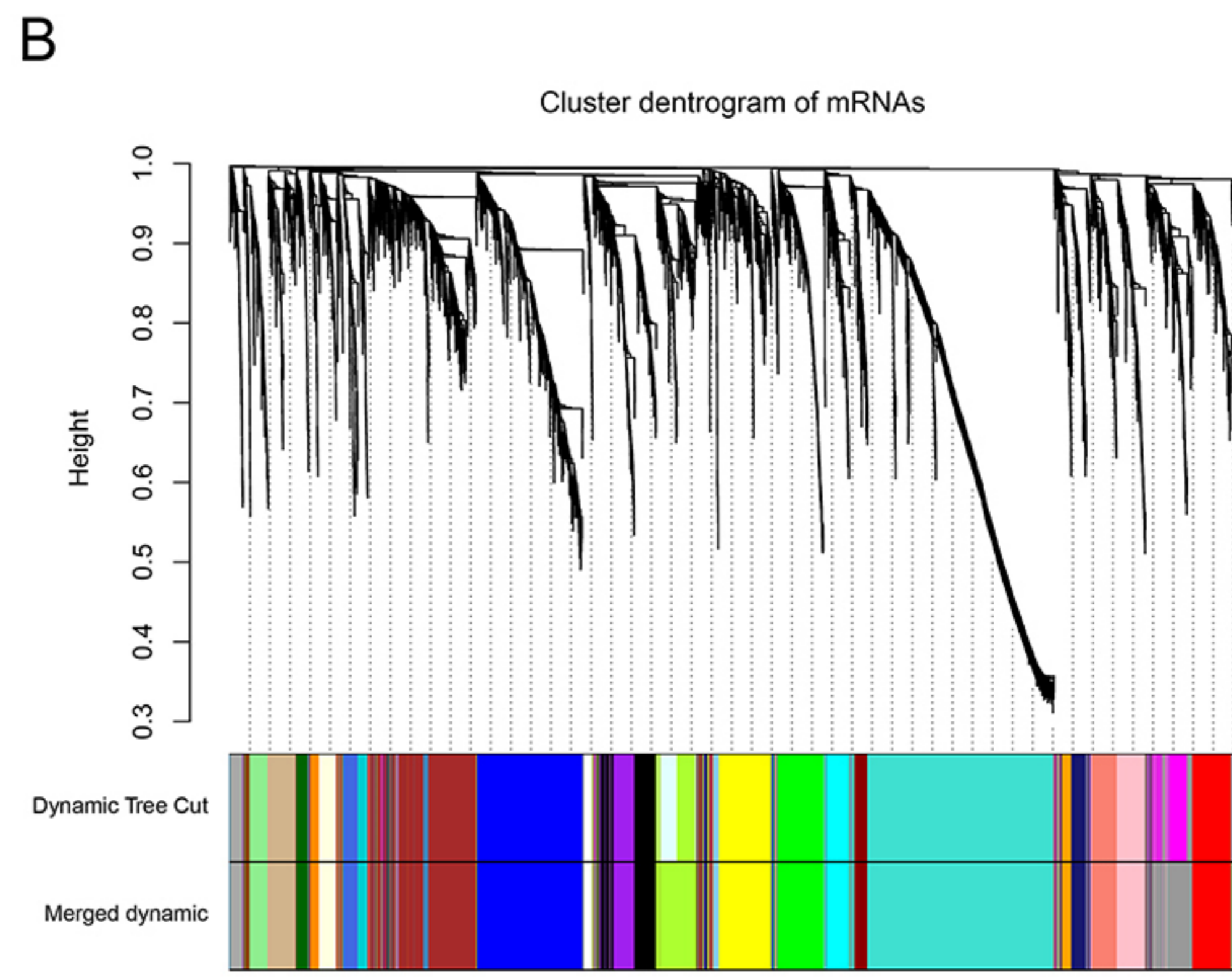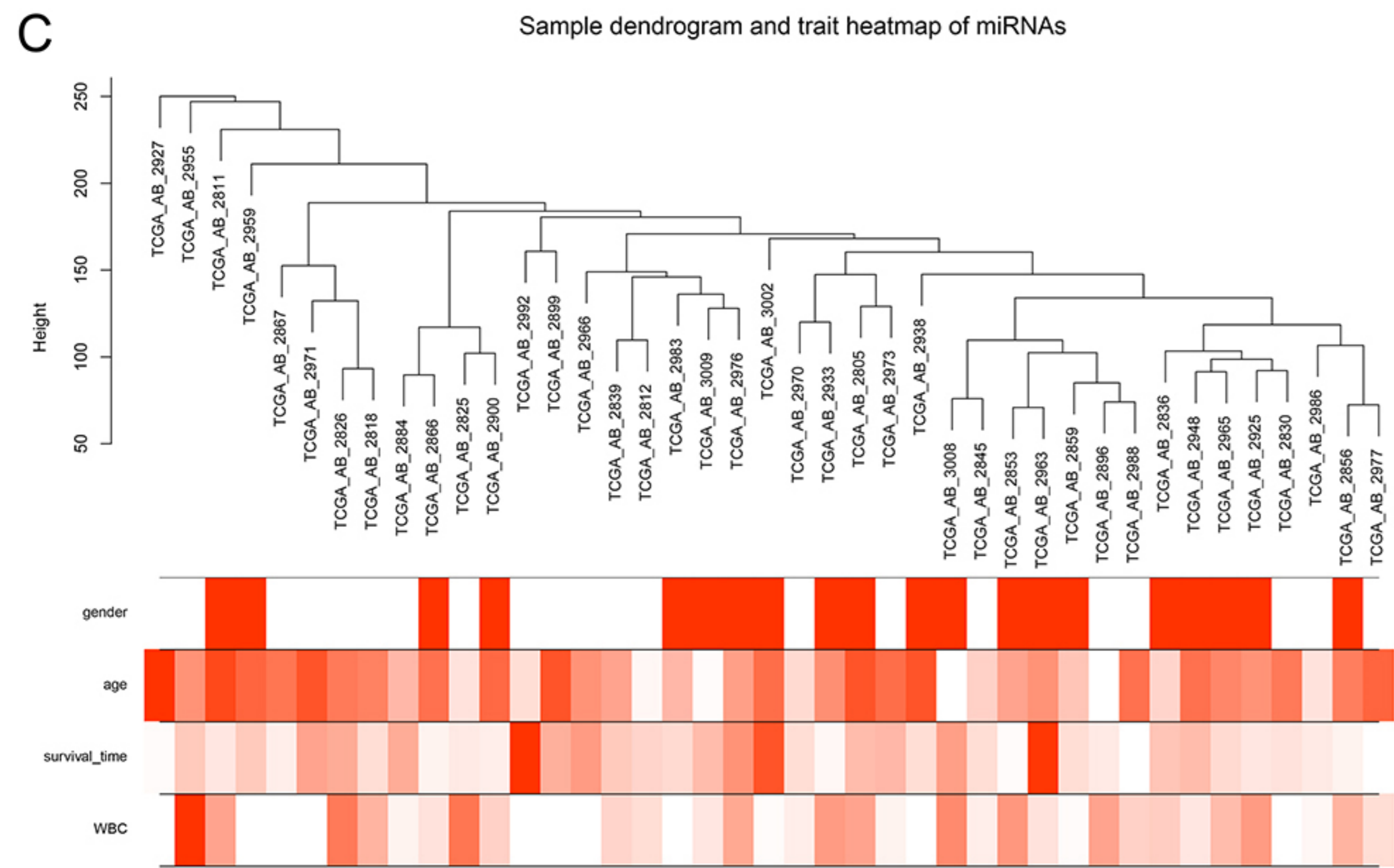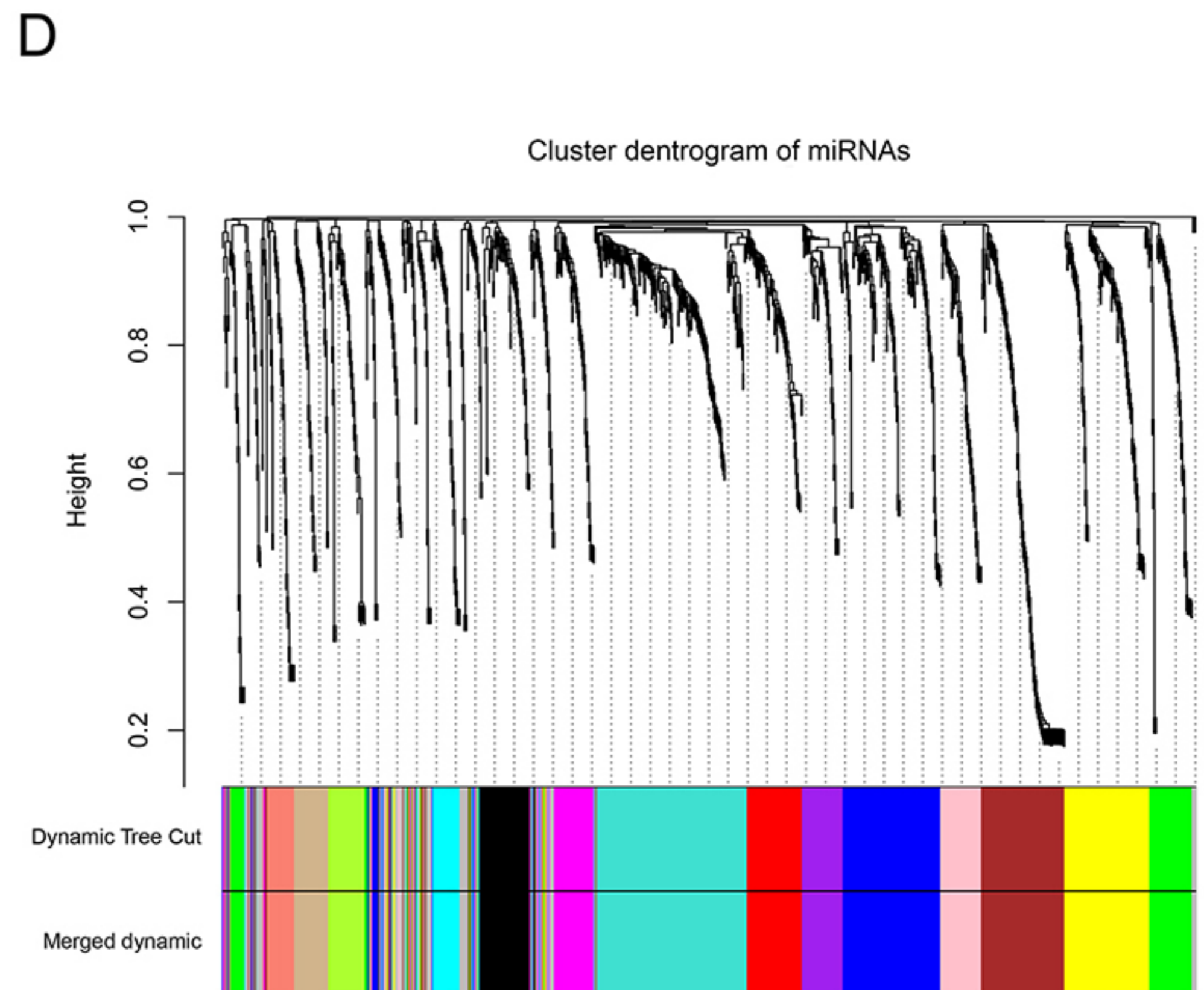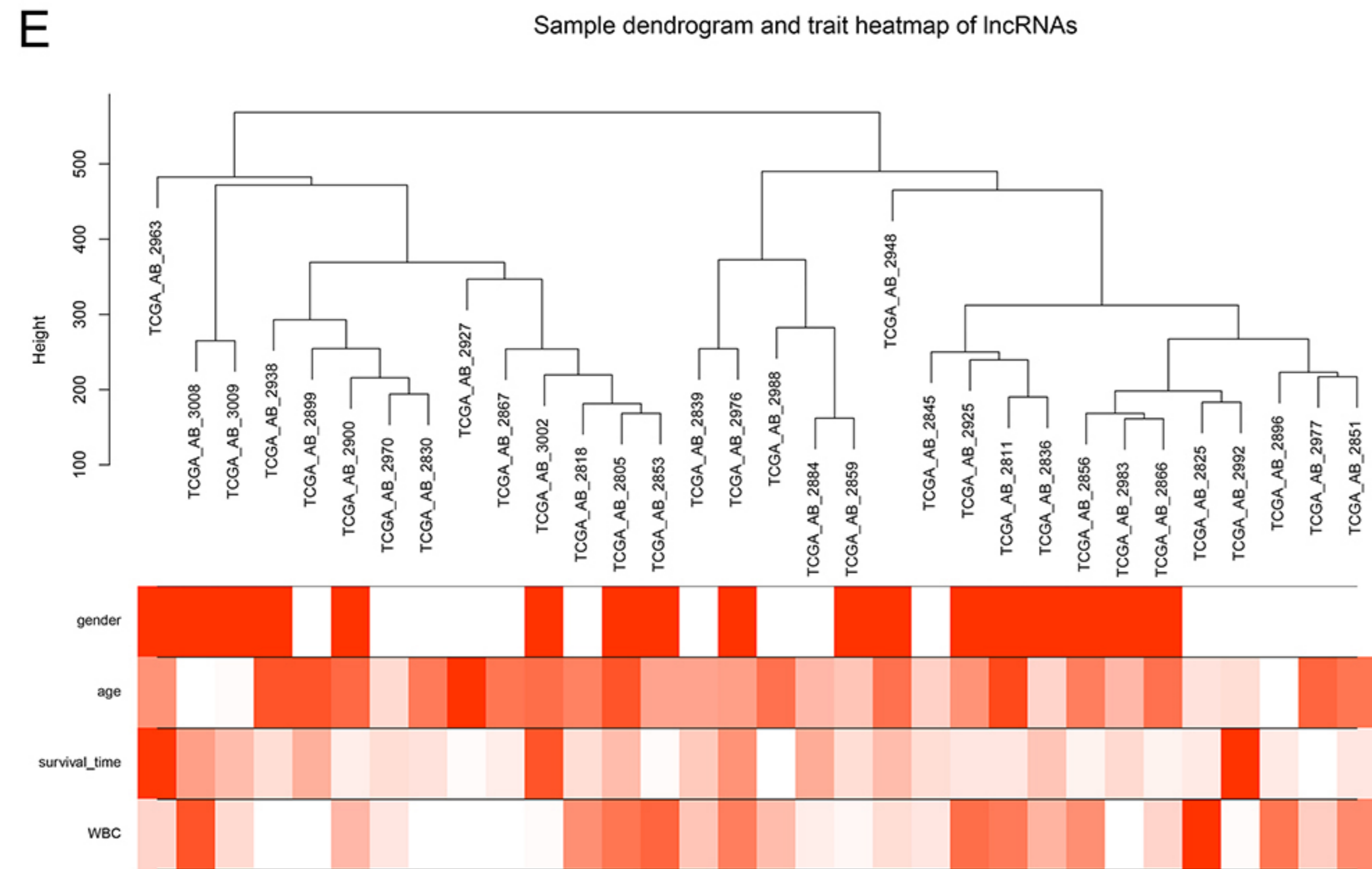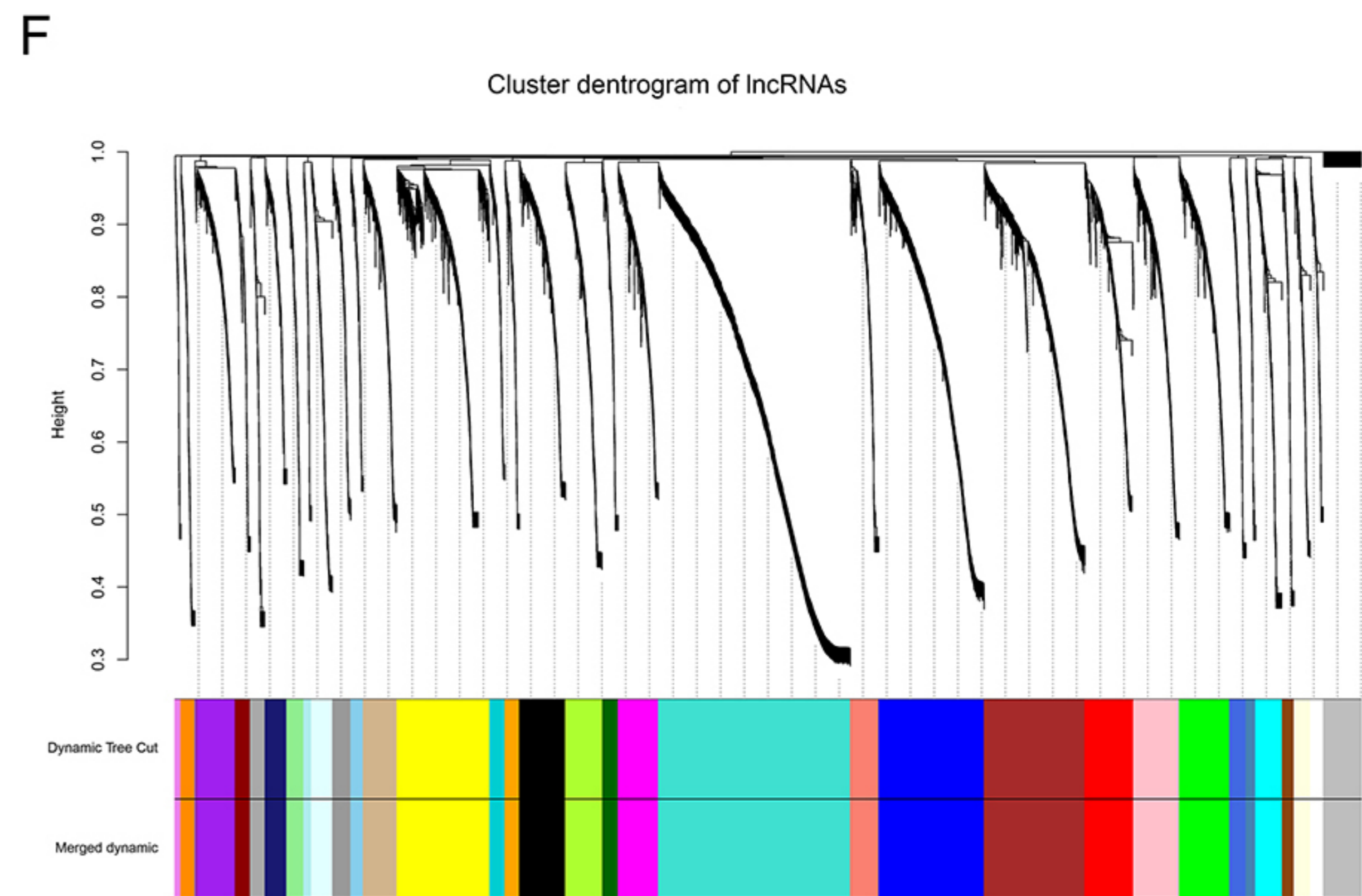

Supplement: Supplementary 2 — Figure 2S: relationship between clinical traits and sample dendrogram, based on the expression data of mRNAs (A), miRNAs (C), and lncRNAs (E). Clustering dendrograms of mRNAs (B), miRNAs (D), and lncRNAs (F). [file 5423694.f2.pdf]

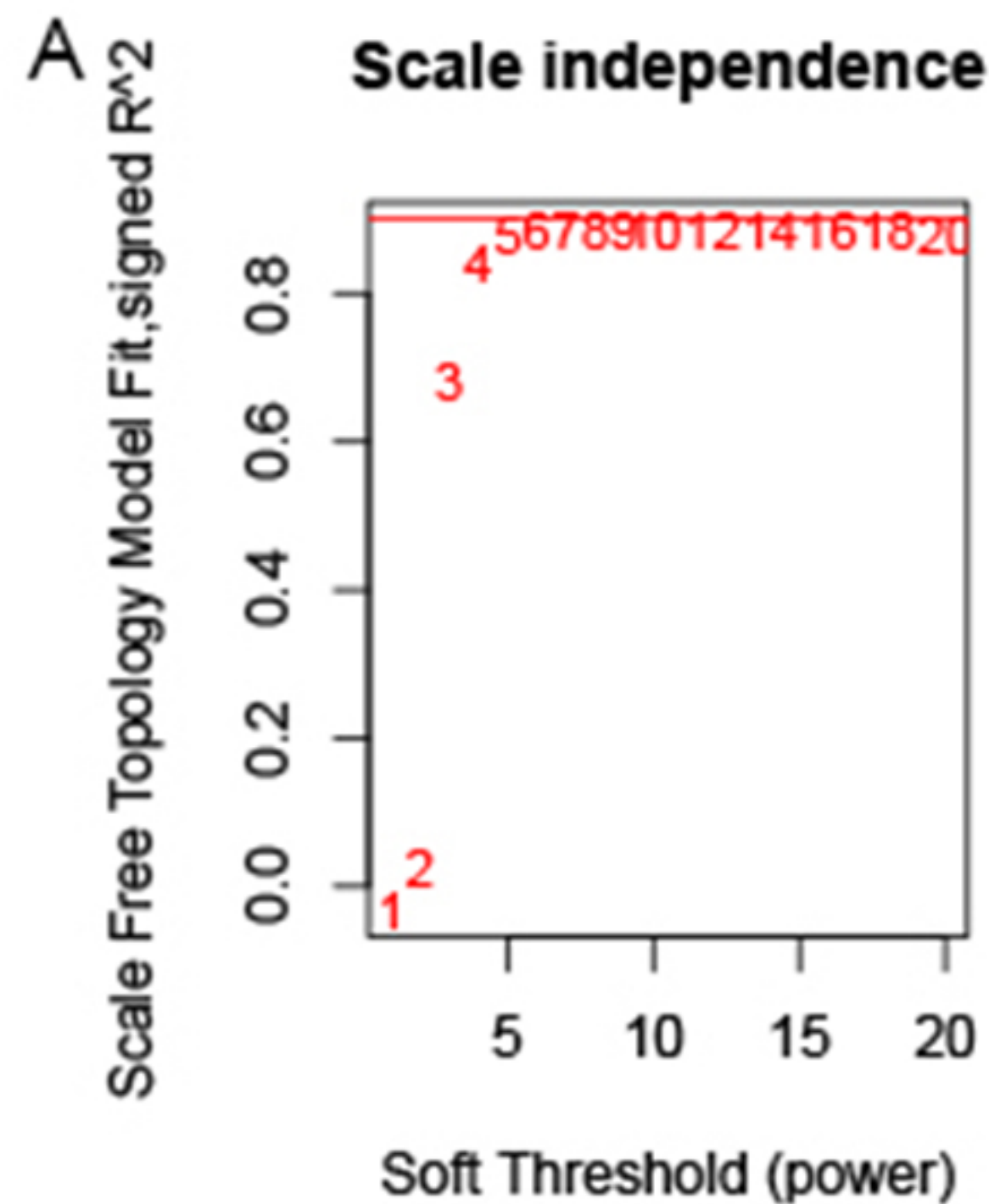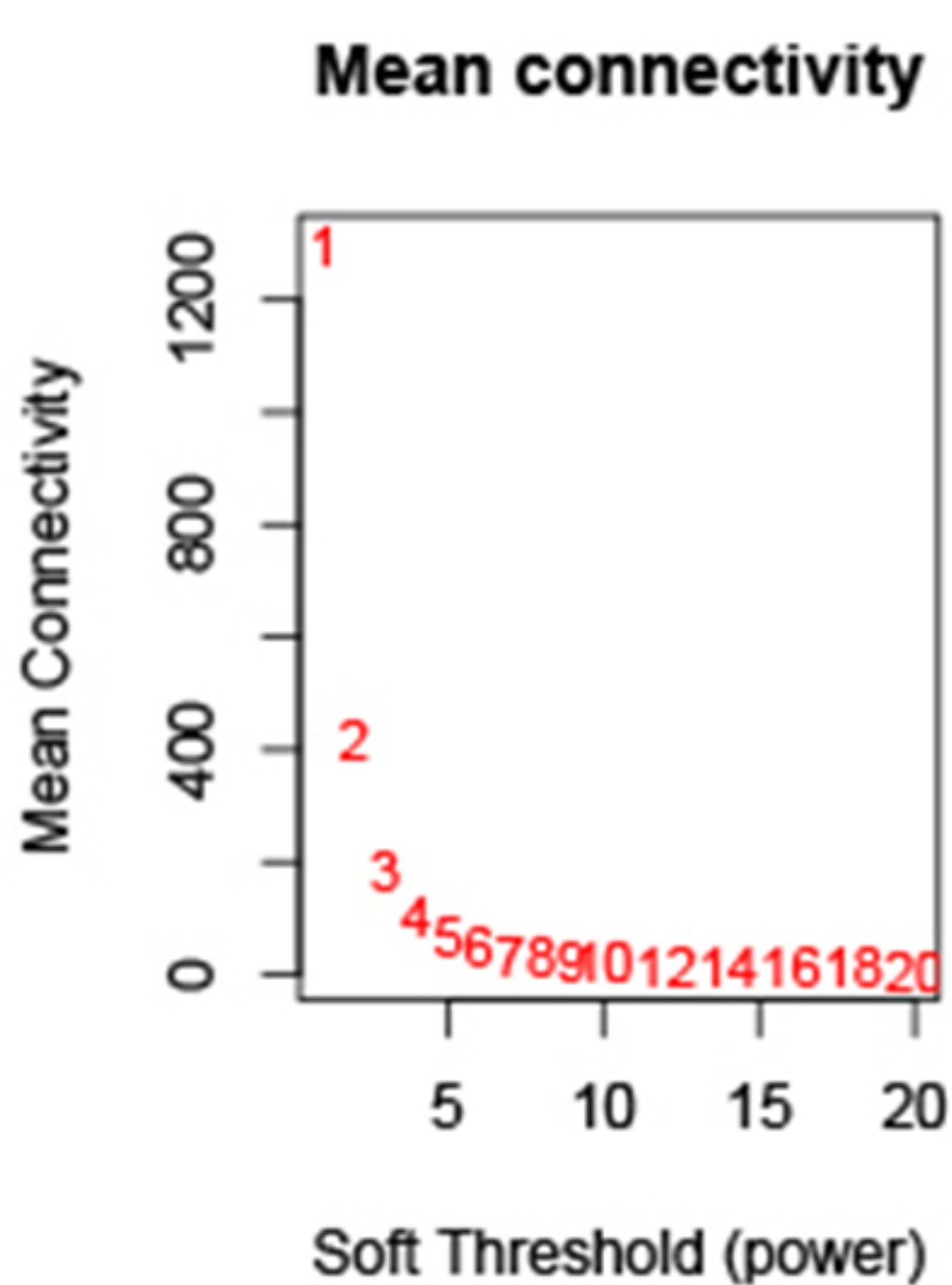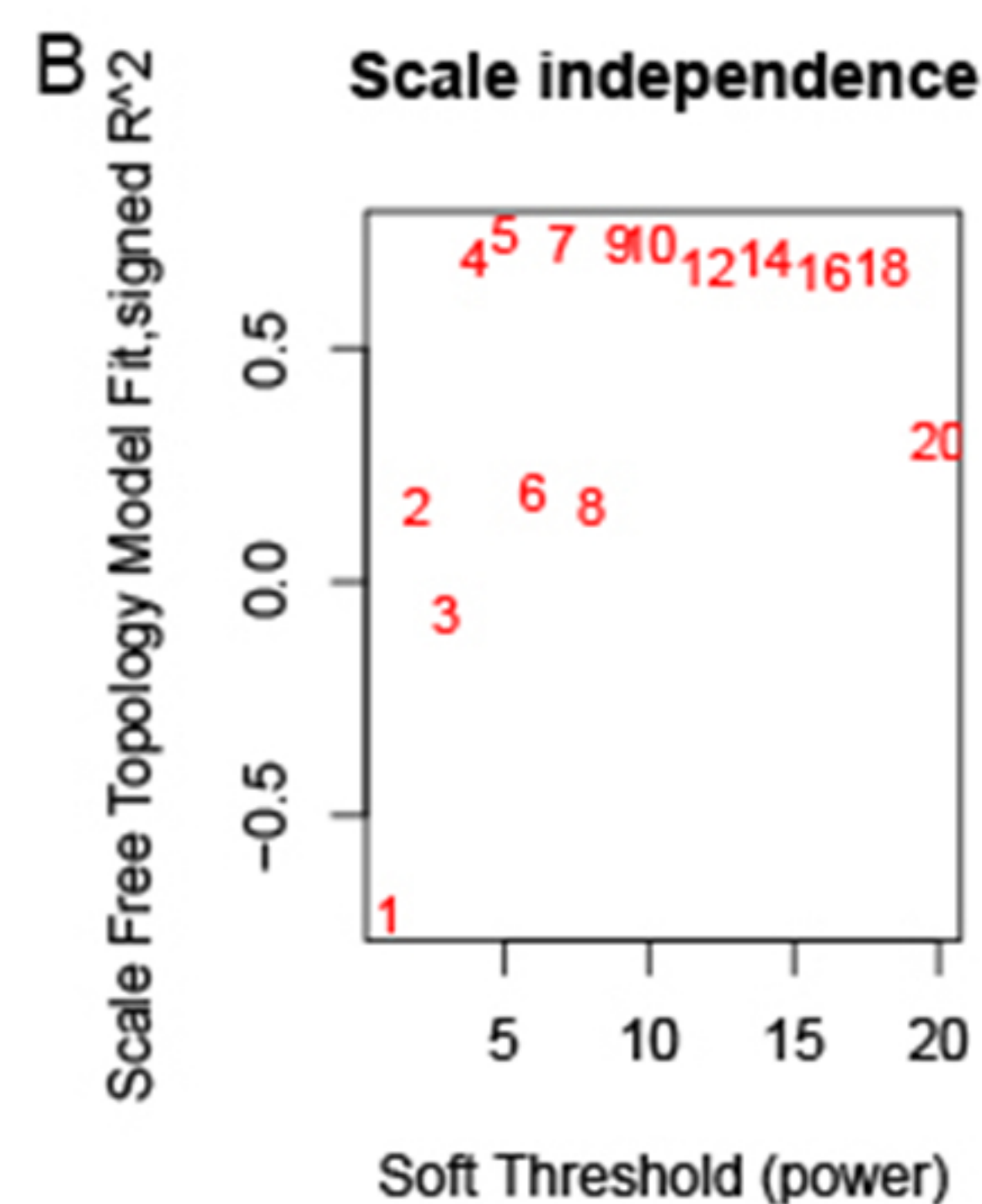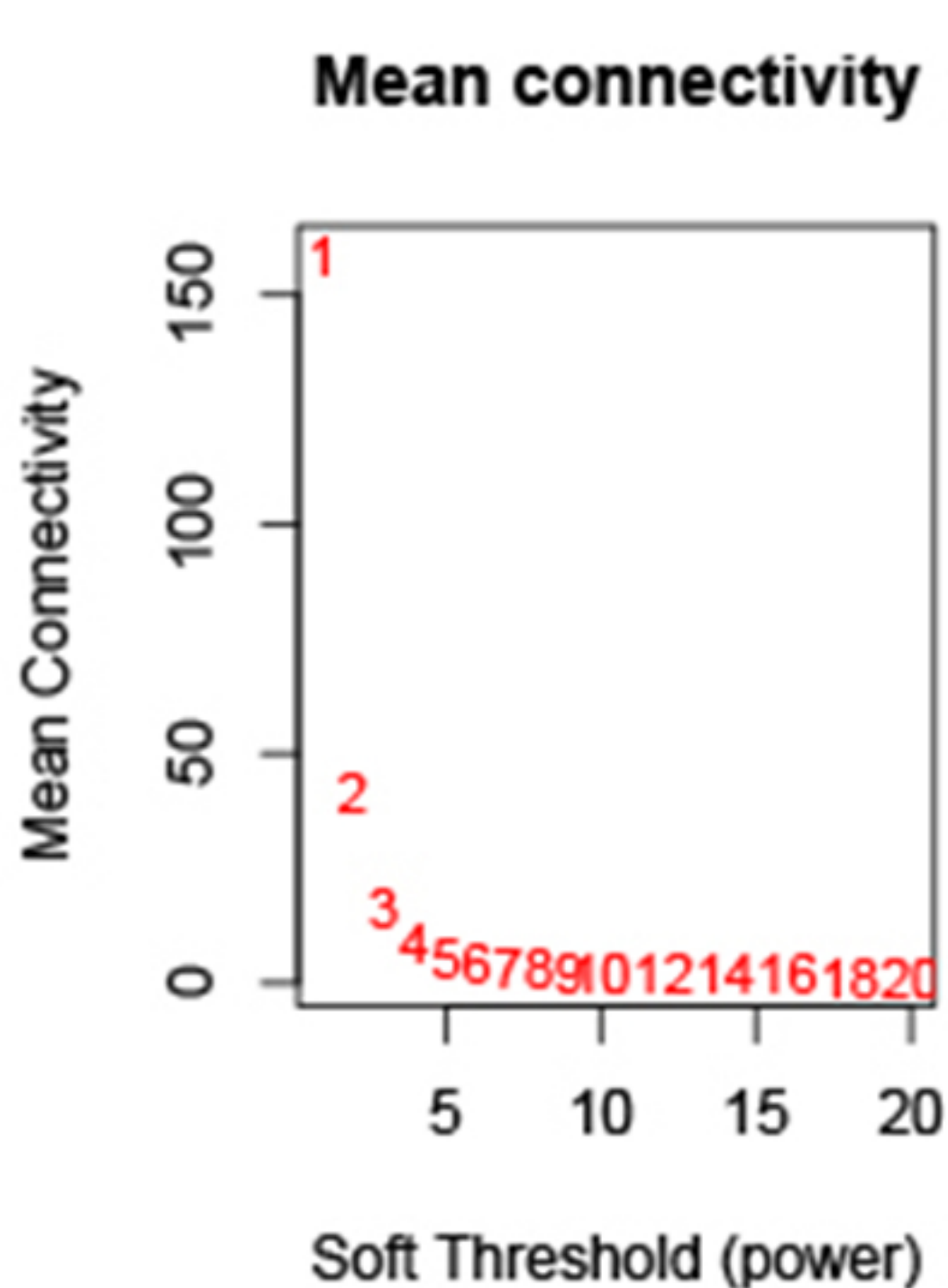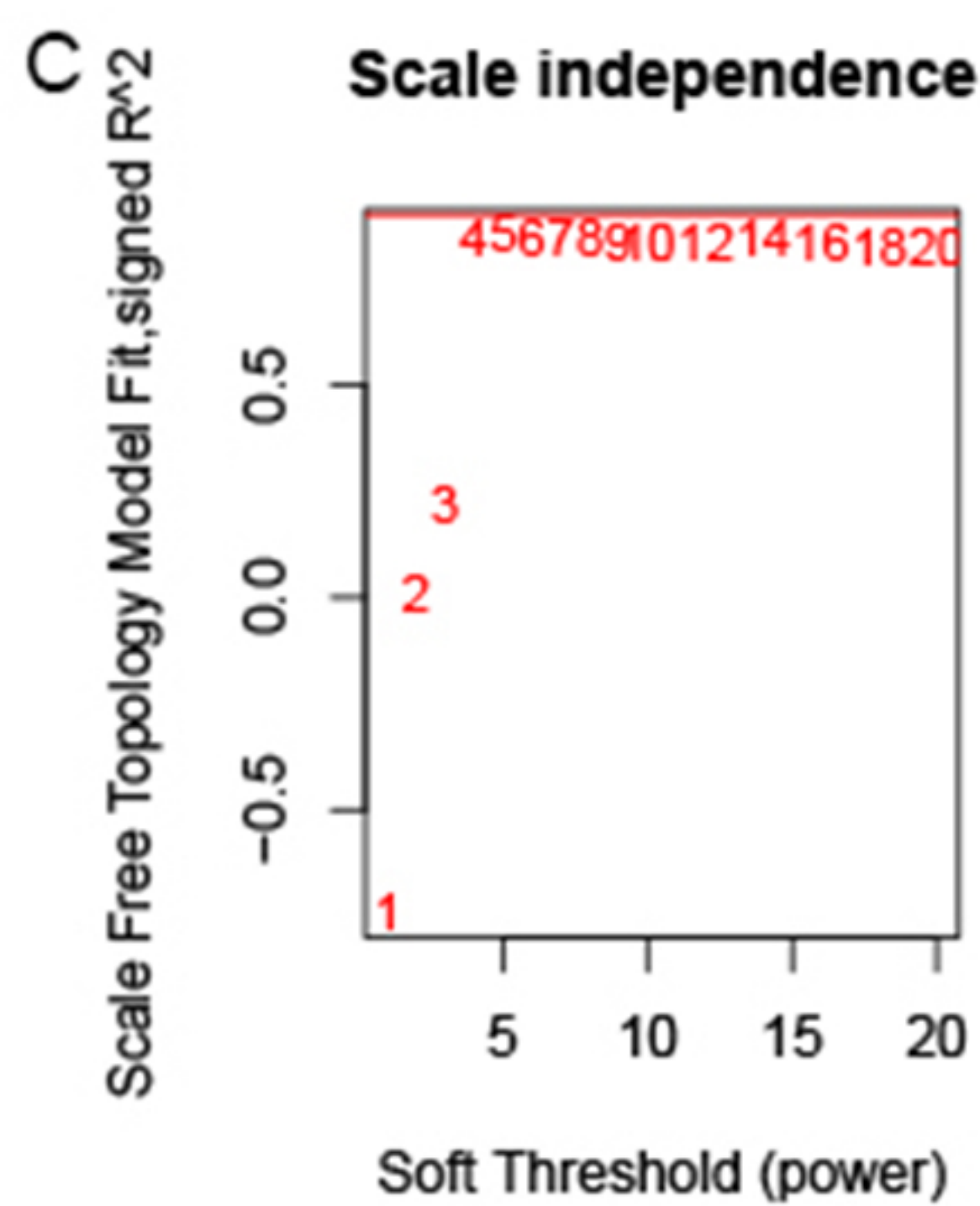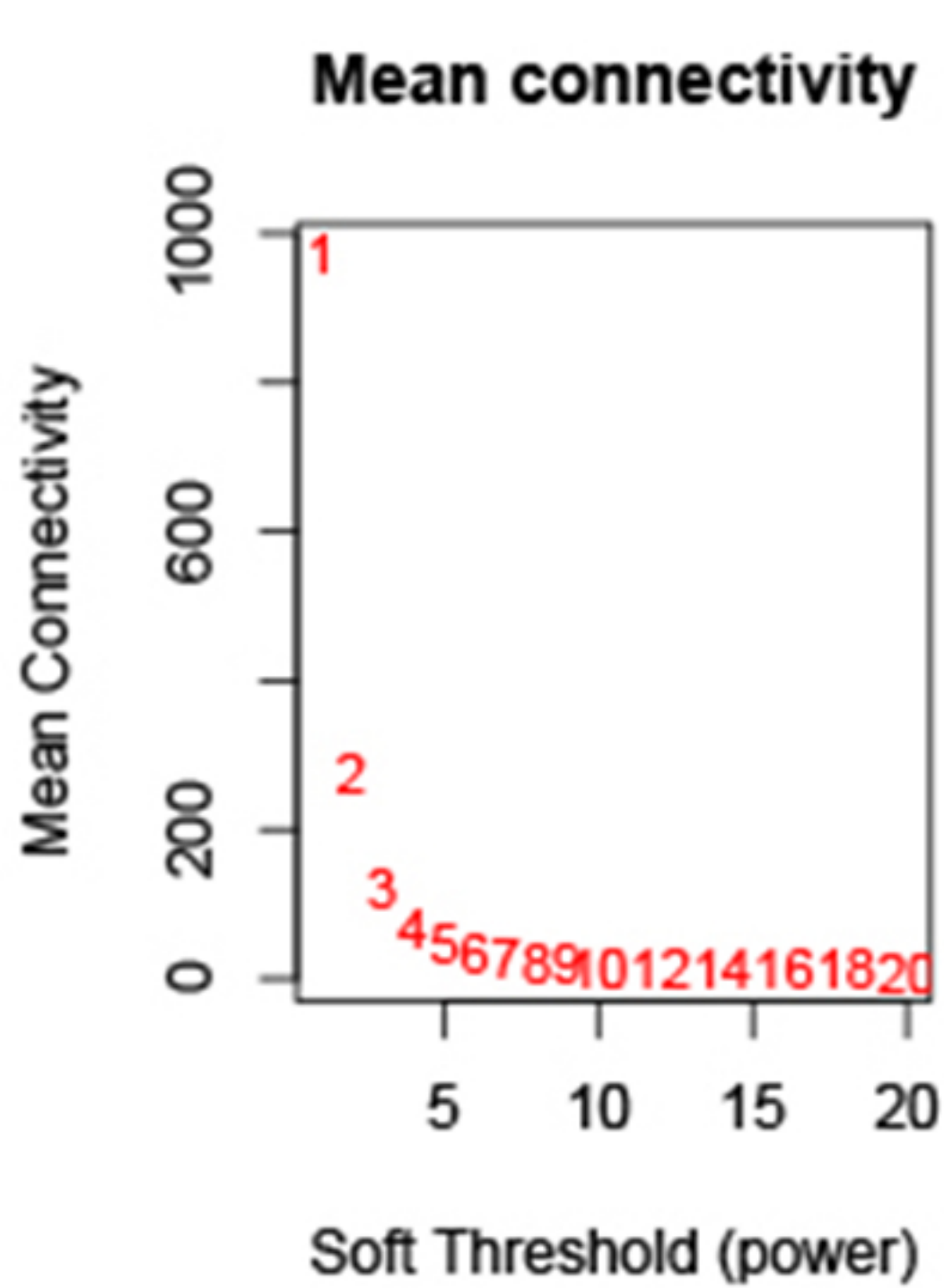

Supplement: Supplementary 3 — Figure 3S: analysis of network topology for various soft-thresholding powers in mRNAs (A), miRNAs (B), and lncRNAs (C). [file 5423694.f3.pdf]

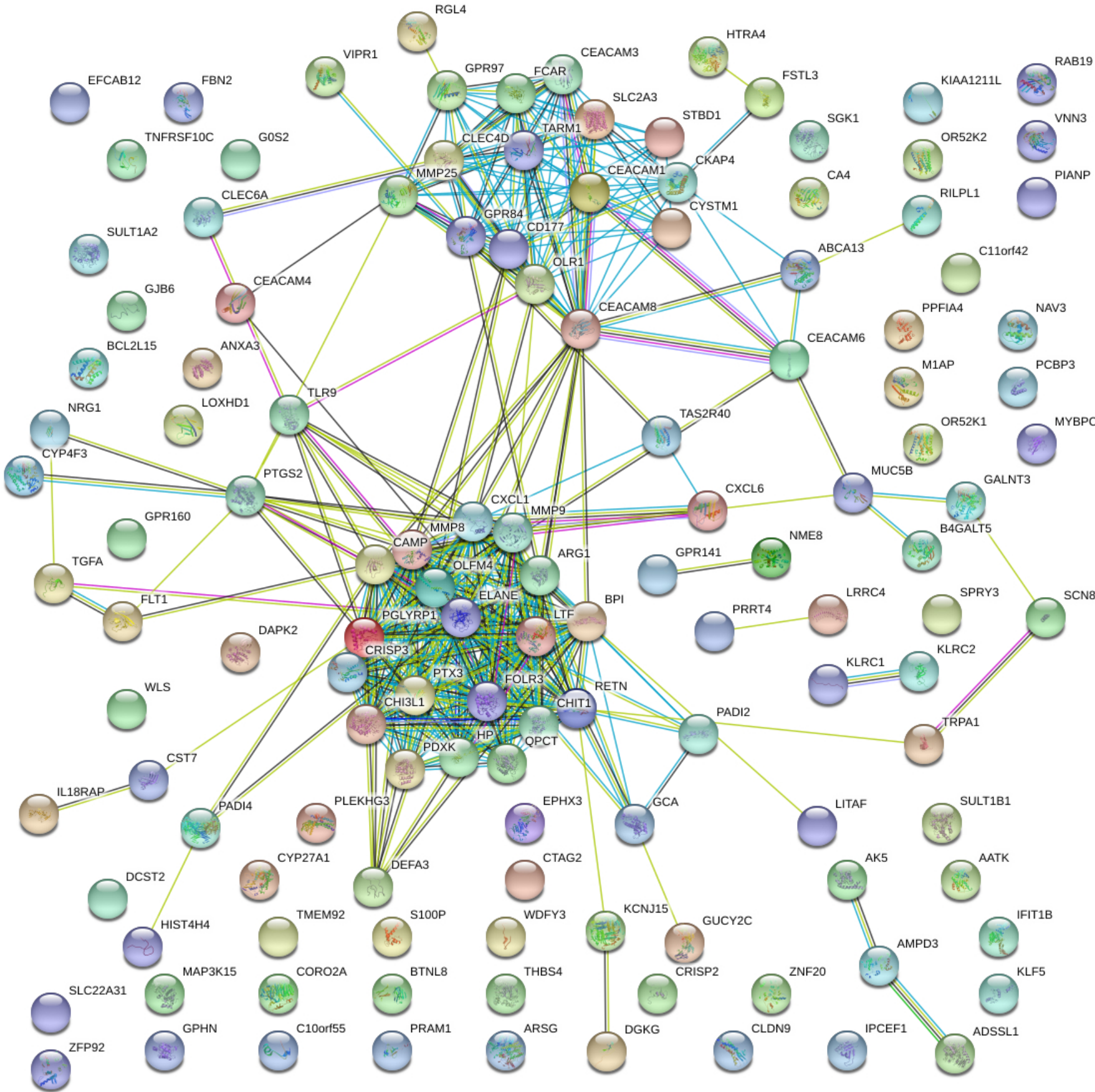

Supplement: Supplementary 5 — Figure 5S: the protein-protein interaction (PPI) network of mRNAs in ME1. [file 5423694.f5.pdf]

A

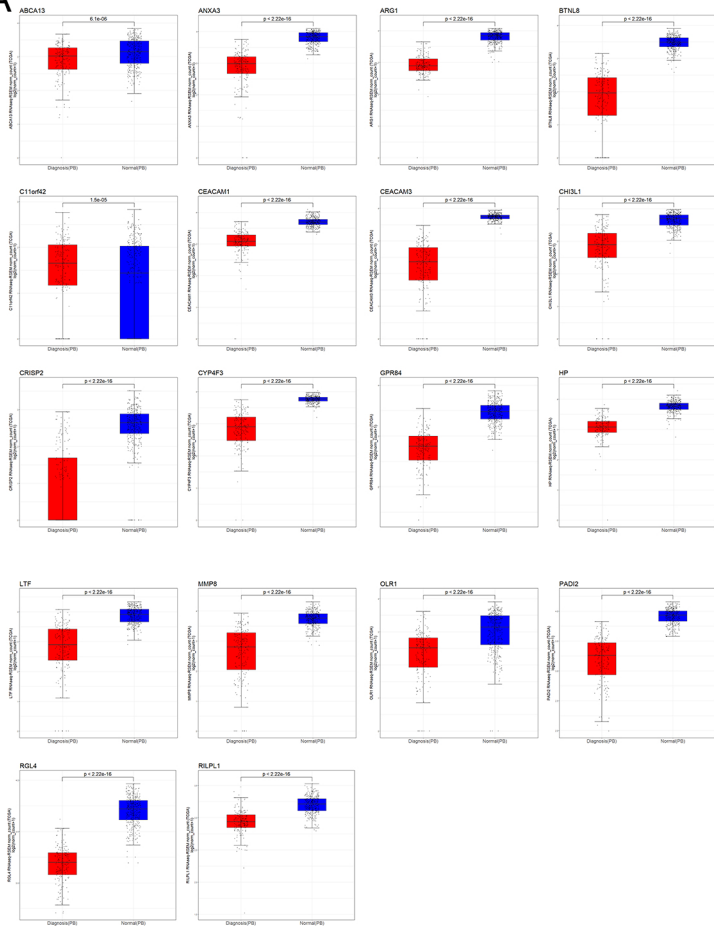

B

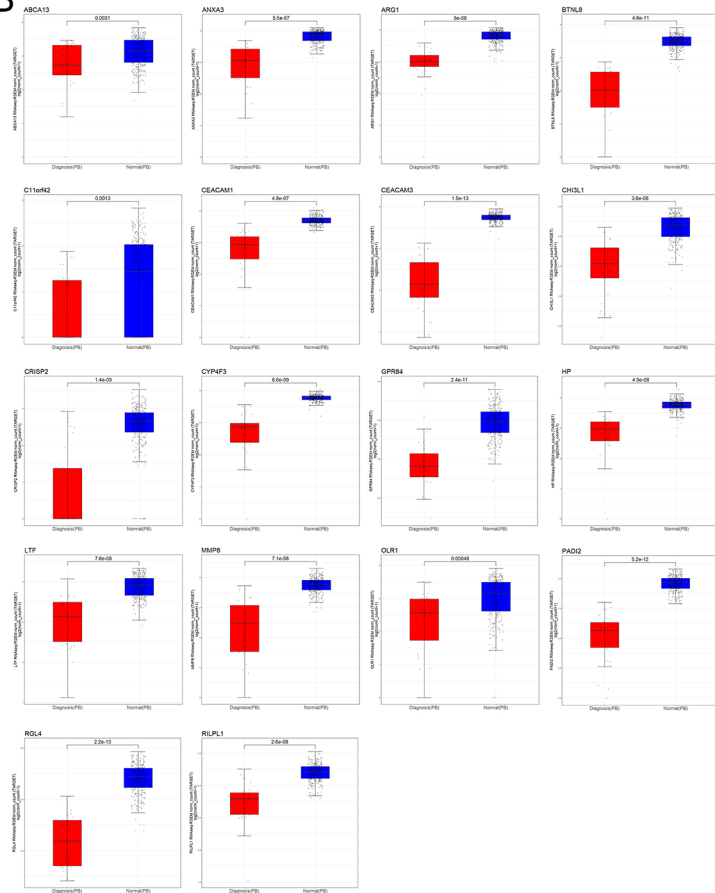

Supplement: Supplementary 6 — Figure 6S: analyses of hub gene expression levels in primary AML PB samples from diagnosis stage and healthy whole blood samples. [file 5423694.f6.pdf]
