# Supplementary material for: Identification of Survival-Related Genes in Acute Myeloid Leukemia (AML) Based on Cytogenetically Normal AML Samples Using Weighted Gene Coexpression Network Analysis
Source: Dis Markers. 2022 Sep 29;2022:5423694. doi: 10.1155/2022/5423694 (PMC9537620; doi:10.1155/2022/5423694)
Supplement: Supplementary 4 — Figure 4S: scatterplots of GS for survival time vs. MM in selected survival-specific module ME1 (A), ME2 (B), ME3 (C), and ME4 (D). [file 5423694.f4.pdf]

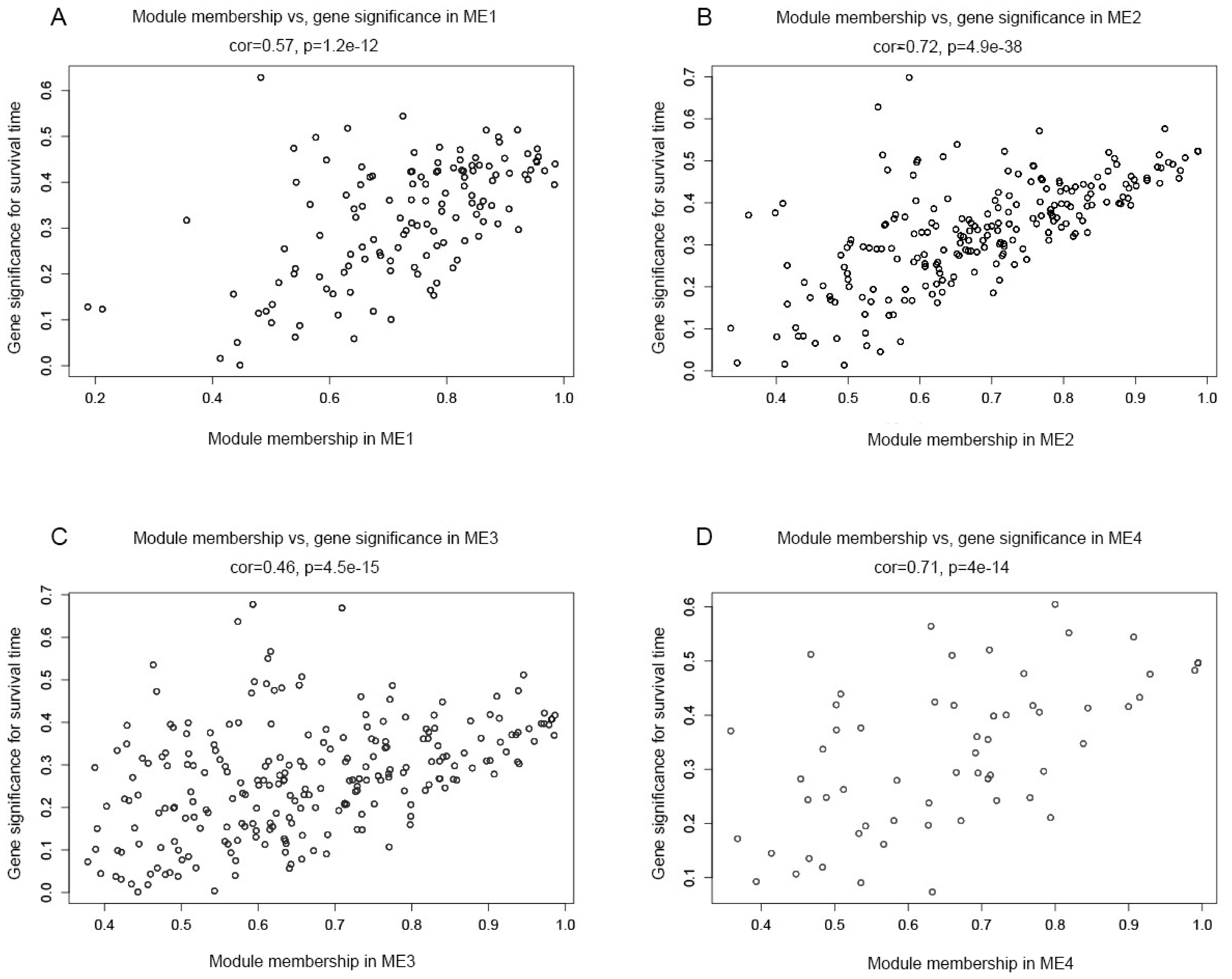
A
Module membership vs, gene significance in ME1
cor=0.57, p=1.2e-12
Gene significance for survival time
Module membership in ME1
B
Module membership vs, gene significance in ME2
cor=0.72, p=4.9e-38
Gene significance for survival time
Module membership in ME2
C
Module membership vs, gene significance in ME3
cor=0.46, p=4.5e-15
Gene significance for survival time
Module membership in ME3
D
Module membership vs, gene significance in ME4
cor=0.71, p=4e-14
Gene significance for survival time
Module membership in ME4
