# Supplementary material for: Identification of Survival-Related Genes in Acute Myeloid Leukemia (AML) Based on Cytogenetically Normal AML Samples Using Weighted Gene Coexpression Network Analysis
Source: Dis Markers. 2022 Sep 29;2022:5423694. doi: 10.1155/2022/5423694 (PMC9537620; doi:10.1155/2022/5423694)
Supplement: Supplementary 1 — Figure 1S: outlier sample removal for mRNAs (A), miRNAs (B), and lncRNAs (C) WGCNA. [file 5423694.f1.pdf]

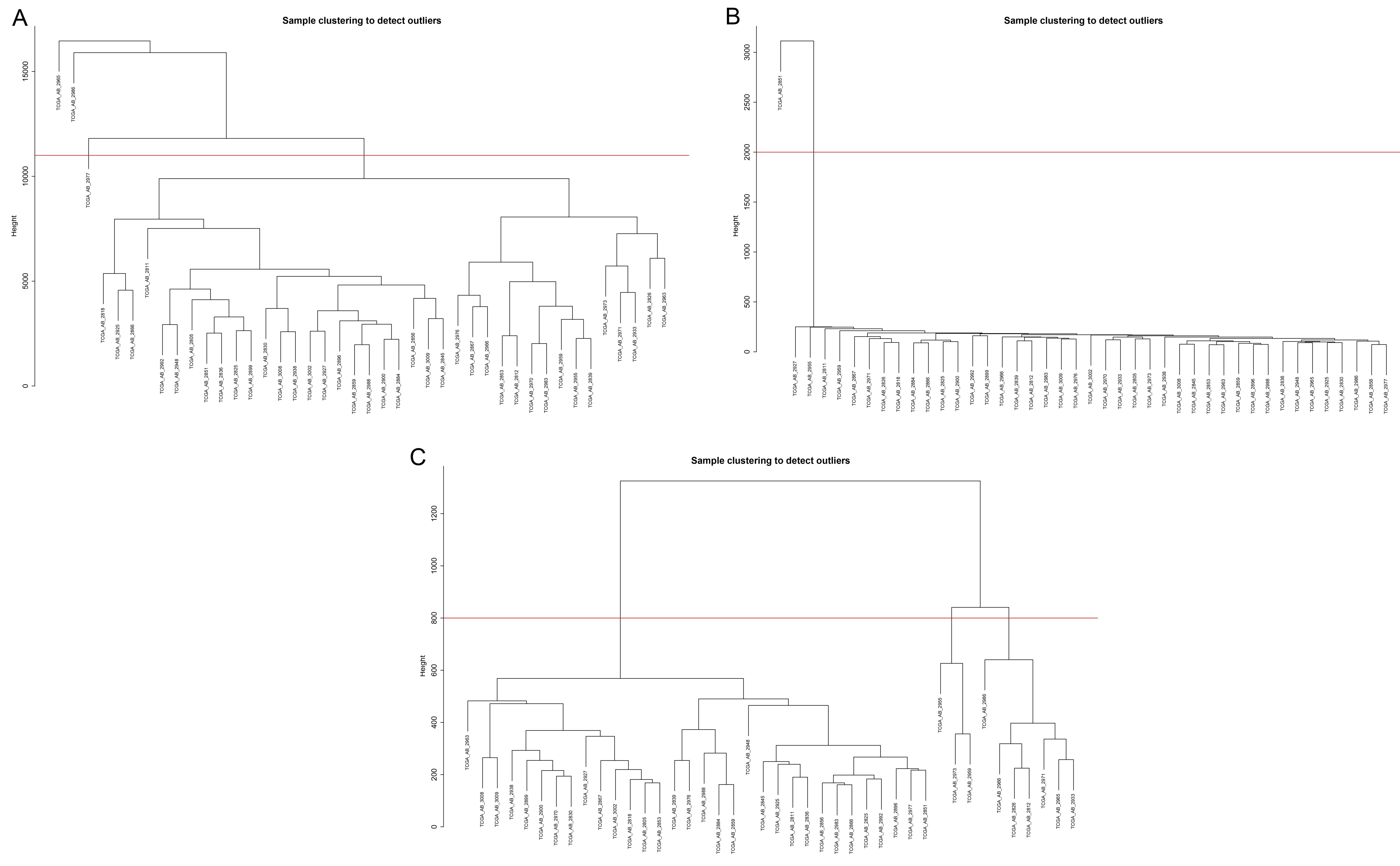

A
Sample clustering to detect outliers
Height
B
Sample clustering to detect outliers
Height
C
Sample clustering to detect outliers
Height
